# Ethnic Background and Genetic Variation in the Evaluation of Cancer Risk: A Systematic Review

**DOI:** 10.1371/journal.pone.0097522

**Published:** 2014-06-05

**Authors:** Lijun Jing, Li Su, Brian Z. Ring

**Affiliations:** 1 Institute of Genomic and Personalized Medicine, College of Life Science and Technology, Huazhong University of Science and Technology, Wuhan, Hubei, China; 2 Key Laboratory of Molecular Biophysics of Ministry of Education, College of Life Science and Technology, Huazhong University of Science and Technology, Wuhan, Hubei, China; University of Kansas Medical Center, United States of America

## Abstract

The clinical use of genetic variation in the evaluation of cancer risk is expanding, and thus understanding how determinants of cancer susceptibility identified in one population can be applied to another is of growing importance. However there is considerable debate on the relevance of ethnic background in clinical genetics, reflecting both the significance and complexity of genetic heritage. We address this via a systematic review of reported associations with cancer risk for 82 markers in 68 studies across six different cancer types, comparing association results between ethnic groups and examining linkage disequilibrium between risk alleles and nearby genetic loci. We find that the relevance of ethnic background depends on the question. If asked whether the association of variants with disease risk is conserved across ethnic boundaries, we find that the answer is yes, the majority of markers show insignificant variability in association with cancer risk across ethnic groups. However if the question is whether a significant association between a variant and cancer risk is likely to reproduce, the answer is no, most markers do not validate in an ethnic group other than the discovery cohort’s ancestry. This lack of reproducibility is not attributable to studies being inadequately populated due to low allele frequency in other ethnic groups. Instead, differences in local genomic structure between ethnic groups are associated with the strength of association with cancer risk and therefore confound interpretation of the implied physiologic association tracked by the disease allele. This suggest that a biological association for cancer risk alleles may be broadly consistent across ethnic boundaries, but reproduction of a clinical study in another ethnic group is uncommon, in part due to confounding genomic architecture. As clinical studies are increasingly performed globally this has important implications for how cancer risk stratifiers should be studied and employed.

## Introduction

The incidence, prevalence and mortality of many cancers among different ethnic populations are often very distinct [1]–[4]. For example, African-American men have among the highest incidence of prostate cancer, while Japanese men living in Japan have the lowest incidence [5]. Strong ethnic differences have also been observed in breast cancer risk; Hispanic and Native American women have a markedly lower incidence of breast cancer compared with non-Hispanic women of European descent [6]. The causes of these disparities are manifold, including intrinsic differences, i.e., genetic variation, and extrinsic differences, which include dissimilarities in social, economic, and geographical environments. Understanding these differences in cancer risk and the underlying causes of these differences is crucial for creating research and health care practices that can span ethnic boundaries.

Genetic variation is an important contributor of cancer risk; and recently genome-wide association studies (GWAS) in several cancers have elucidated the roles of many common risk alleles in affecting disease susceptibility. BRCA1 and BRCA2 are the most well-known genes whose mutations are linked to breast cancer risk, and the list of known risk alleles is rapidly expanding [7]–[10]. It is becoming increasingly apparent that ethnic background can play an important role in determining how different alleles are associated with risk of cancer [11], [12]. Furthermore, several studies examining factors contributing towards cancer susceptibility across multiple ethnic groups, such as the Multi-Ethnic Cohort (MEC) Study, have shown that the tested non-genetic factors did not account for all differences in cancer susceptibility among ethnic groups [13]. In a large prospective study of colon cancer, one MEC study found that ethnic variation in the incidence of colon cancer was not fully explained by differences in the prevalence of the tested extrinsic risk factors: Japanese Americans of both sexes and African American women remained at increased risk of cancer relative to those of European descent after accounting for differences in tested extrinsic risk factors [14]. Similarly, another MEC study found significant differences in the association between cigarette smoking and the risk of lung cancer among five ethnic groups. The findings could not be explained by differences between populations in the tested risk factors, including diet, occupation, and socioeconomic status [15]. These studies suggest that unexplained genetic factors may be important for understanding differences in cancer risk between ethnic groups.

Genetic variation among ethnic groups impacts cancer risk in multiple manners: there may be different frequencies for a risk allele between populations, an allele may have dissimilar associations with risk in different populations, and an allele may interact with other genetic or environmental factors that vary among populations. The HapMap project has made great advances in elucidating the varying prevalence of alleles among ethnic groups [16]. However, information about the other ways in which differences among ethnic groups can affect cancer susceptibility is less well systematically studied. While the Multiethnic Cohort Study is an example of how this research can occur, and individual studies highlight the importance of an understanding of ethnic variation, there is a pressing need for more thorough surveys of the interplay of genetics and ethnicity in determining cancer susceptibility.

As the potential clinical utility of risk alleles for patient stratification are increasingly considered [17]–[19], the need to understand how these variations may differentially affect members of diverse ethnic groups is growing. Concomitantly, the accurate translation of clinical studies from one ethnic group to another becomes more important as economic factors drive an increasing number of clinical studies to be performed as multiregional trials, with global results used in support of applications in the sponsoring country [20], [21]. To date, well-populated studies for the identification of associations between gene variants, and the validation of these associations, has been conducted primarily in populations of European ancestry; however the utilization of these findings in other populations may not be straightforward. A study by Ioannidis et al., which examined published meta-analyses of gene association studies involving several complex diseases (including four cancer types) wherein the polymorphism was seen to be significant in at least one ethnic group, found low heterogeneity among ethnic groups in the majority of the studied loci [22]. This study, which focused on validation studies of candidate markers, possibly contained many causative genetic variants, and suggests that basic biology is conserved across ethnic boundaries. However, many apparent differences between ethnic populations in how alleles are associated with cancer risk have been identified (for examples, Table S2–S7). A related study to the 2004 study of Ioannidis et al. showed that when loci identified from genome wide association studies of several complex diseases were assessed the majority of studied loci did not show consistency of disease association across ethnic backgrounds [23]. This second study, focusing on GWAS nominated variants, likely includes many markers only in linkage disequilibrium with the causative variant. Similarly, a study of several GWAS identified prostate cancer risk loci showed that most of the assessed loci did not replicate in a Japanese population [24]. The results of these study suggest that GWAS identified loci, as compared to those identified from family studies (such as BRCA1) or candidate gene approaches, are less likely to be tightly linked to the true functional loci, leading to relatively weaker strengths of association. Further clarification of the role of ethnic background in affecting the association of variants with cancer risk is needed. As cancer risk profiling becomes increasingly common, and as an increasing number of treatment decisions are linked to genotyping results, e.g., erlotinib used for the treatment of lung cancer patients with EGFR mutations [25], or cetuximab therapy for colon patients lacking KRAS mutations [26] elucidating the roles ethnic differences have in the clinical management of cancer will entail a better understanding the relationship between ethnicity and predictive markers.

Here we present a survey and systematic analysis of association studies conducted in multiple ethnic groups for the primary known risk alleles in lung, stomach, liver, colon, breast and prostate cancer. These cancers were chosen based on incidence rates; lung, stomach, liver, colon and breast cancer are the cause of most cancer-related deaths each year in both sexes, and in men the second most frequent cause of cancer-related mortality is prostate cancer [27]. We find that most of the associations between gene variants and cancer risk that we surveyed did not validate in new ethnic populations, consistent with other studies that have examined the reproducibility of complex disease risk variants. As low prevalence of the risk alleles in some populations may lead to studies being inadequately populated to validate associations found to be significant in another population, some of the disparate associations among ethnic groups may be attributable simply to low powered studies. However we found that, though many studies were inadequately powered, low allele frequency did not explain the inability to reproduce significant findings between ethnic groups. Instead, we show that differences in linkage disequilibrium appear to be associated with differences in the odds ratio (OR) between ethnic groups. Despite the infrequent validation of significant associations, we find that variability in the odds ratios for the studied variants among ethnic groups are usually not significant. This suggest that the basic biological role, or at least their association, of genetic variants are broadly consistent across ethnic boundaries, but that most well-studied risk loci may be poorly linked to the probable true functional loci in many populations. Therefore great attention needs to be paid when attempting to translate cancer risk associations between ethnic groups. Identification of more tightly linked risk markers is important, as well as validation within the ethnic group in question, for understanding the potential role of ethnic background in affecting cancer susceptibility and to allow proper utilization of potentially clinically relevant findings between ethnic groups.

## Materials and Methods

### Search Strategy

We systematically searched PubMed (http://www.ncbi.nlm.nih.gov/pubmed/) and Web of Science electronic databases (http://apps.webofknowledge.com) for meta-analyses published prior to December 2013 that reported the association between alleles and cancer risks within ethnic groups in six cancer types: lung, stomach, liver, colon, breast and prostate. We also searched for SNPs currently used by major popular genome profiling services for the risk stratification of the six cancers, including 23&Me (https://www.23andme.com/), Navigenics (http://www.navigenics.com/), and United Gene (http://www.ugi.hk/), for these alleles we broadened the search to any study (not limited to meta-analysis) that provided information on ethnic background. This was to ensure that variations already used in commercial assays were in this study; however the records found in these specific searches were all identified by the searches open to all variants. When multiple reports were available for a single study, only the most recent report was included.

### Inclusion Criteria

For inclusion, the studies must have met all the following criteria: (1) included information for at least two ethnic groups; (2) were meta-analyses of case-control or cohort studies that had original data of a quantitative assessment of the relationship of one gene or SNP and risk of one of the six specified cancer; (3) results were expressed as an odds ratio; and (4) with a 95% confidence interval (CI) for the OR. In addition, variants included in commercial personal genomics assays offered by 23&Me, Navigenics, and United Gene to estimate the risk of the six cancers were used as search criteria, the requirement of the study being a meta-analysis was not used for these variants.

### Exclusion Criteria

The following exclusion criteria were used: (1) case-only studies, case reports, editorials and abstracts; (2) studies that were missing case and control numbers or an OR; and (3) studies reporting only results in only one ethnic group. No language or publication date restrictions were imposed.

### Statistical Analysis

Data from all included papers was tabulated (Tables S1–S7). When data for multiple genetic models are presented, the model with the largest population that had a significant association between allele and risk was selected for further analysis. If no significant association existed then the model with the largest total population was selected. In tabulating all pairwise comparisons between ethnic groups for each SNP, the ethnic group with the largest population giving a significant result was selected as a reference population. When significance was not found for any ethic group the largest population was used as the reference. For alleles where one ethnic group exhibited a significant association with cancer occurrence and another group did not, a power analysis was performed for the non-significant studies using the Genetic Power Calculator [28], based on the cancer’s prevalence in the relevant ethnic groups, the number of cases and controls, and an estimated relative risk. Prevalence was derived from World Health Organization statistics (http://globocan.iarc.fr/)[29]. Relative risk was estimated using the power calculator using the known prevalence and the given odds ratio as an initial approximation of the relative risk. For this study, “well powered” is a power greater than or equal to 80%. To assess heterogeneity among ethnic groups for the associations with risk a Breslow-Day test with Tarone’s adjustment [30] was employed, as implemented in the R metafor package [31]. Loci were excluded if incomplete case and control numbers for each ethnic group were not reported. Pairwise linkage disequilibrium was measured using Haploview 4.2 software [32]. All r^2^ values for SNP pairs with the assessed variant within a region 50 kilobase on each side of the locus of interest were evaluated with a one-way permutation test based on Monte-Carlo resampling (replications = 10,000) to compare LD patterns between ethnic groups, as implemented in the R coin package [33]. Only SNPs which had at least 20 SNP pairs available for the LD analysis within this region were assessed. Agreement between odds ratios was compared with a z test on the difference of the odds ratios 
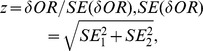
 only the odds ratios for which the association study was significant or were at least 80% powered to validate the significant finding in the reference population were evaluated. A mixed model with the SNP as a grouping variable and cancer type as a random effect were used to evaluate significance of the association of agreement in LD between ethnic groups with significance of the difference in OR. Potential publishing bias was assessed using funnel plots and Egger’s regression test [34]. Results were considered significant for p-values (two tailed) less than 0.05.

## Results

### Genetic Variant Selection

**Figure 1:**
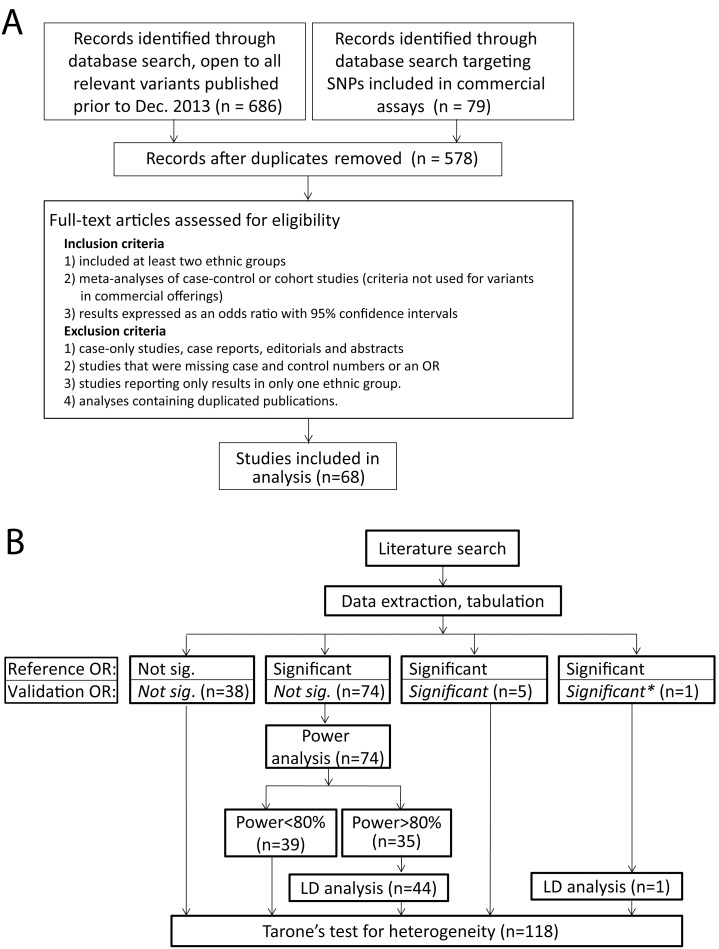
Search strategy and study design A) Literature search strategy. B) Associations between markers and cancer risk were compared between ethnic groups. Among the 86 SNPs assessed in this study, 123 pairwise comparisons of association results between ethnic groups were made. The association results were assessed to determine if each ethnic group was sufficiently populated to find significant results found in other groups. Where differences were found between groups, linkage disequilibrium analysis was performed. The Breslow-Day test for heterogeneity with Tarone’s adjustment was used on all studies with sufficient data. *Both groups had significant results, but with opposite signs.

We searched for studies comparing association of cancer risk with allelic variations in breast, colon, lung, liver, gastric and prostate cancer in different ethnic groups. This analysis was open any genetic variant affecting the six cancer types but we also specifically included SNPs currently used by major popular genome profiling services for the risk stratification of the six cancers. Based on this strategy, 68 publications met our inclusion criteria for further analysis (Figure 1A). We obtained data for 96 assessed associations between cancer risk and genetic variants across the six cancers (82 unique variants) (Tables S1) from these papers. In total, 50 loci were associated with breast cancer, the other SNPs were distributed as: colon: 23 SNPs, liver: 8 SNPs, gastric: 4 SNPs, lung: 6 SNPs and prostate 5 SNPs. The ancestral allele and frequency of ancestral allele are summarized (see Methods, Table S1). Study process is shown in Figure 1B. An assessment of potential publication bias for the included studies (using funnel plots and Egger’s regression test) showed no significant bias for all cancers except breast (Figure S3). When assessed within each ethnic group, no bias was observed in the included breast cancer studies either.

### Association with Cancer Risk among Ethnic Groups

To estimate the importance of genetic contributions among ethnic groups in the evaluation of cancer risk, we surveyed the OR’s primarily in populations of European, Asian and African descent. To reduce the amount of heterogeneity within North American studies (where populations may have diverse ancestry), if the ethnic background was not stated then participants were not assumed to have European heritage. The odds ratio values and numbers of studies, number of cases and controls for each model, and the type of genetic models tested were collected (Tables S2–S7). To clarify the possible causes of the dissimilarities in OR observed between ethnic groups, we calculated the power of all studies within each ethnic group that gave non-significant results where another ethnic group had a significant association for the same allele in the same study. Results are tabulated in table 1 for all pairwise comparisons between ethnic groups within each SNP; the available data for the 82 unique variants assessed in this analysis allowed 123 pairwise comparisons. Disagreement between populations on the presence of a significant association is potentially due to the non-significant study being underpowered. Indeed, within the 80 comparisons between ethnic groups where a significant association was found in at least one population, 39 comparisons (49%) were underpowered to validate the significant result. However, in the 41 comparisons between ethnic groups that were adequately powered to validate the significant result, only 12% (5/41) of allele associations replicated, 85% of comparisons in well-powered studies (35/41) showed no significance for the association in the validation population. The results were similar across all studied cancers and between ethnic groups. In general, the association of genetic loci with cancer risk usually do not replicate in different ethnic groups

**Table 1:** Concordance of association with cancer susceptibility.

Reference Population	Validation Population								
association w/risk	Association w/risk	Power	breast	colon	prostate	gastric	liver	lung	total
s	ns	>80%	21	4	1	1	1	7	35
s	ns	<80%	19	7	6	3	2	2	39
ns	ns	na	25	10	0	0	3	0	38
s	s	na	3	1	1	0	0	0	5
s	s*	na	1	0	0	0	0	0	1
s	nd	nd	1	0	0	0	4	0	5
		**Total**	70	22	8	4	10	9	123

All pairwise comparisons between ethnic groups for each SNP are shown. A reference population was chosen for each SNP as the ethnic group with the largest population giving a significant result, when no significance was found the largest population was used. s, reported association with risk was significant; ns, not significant; na, not applicable; nd, not determined. *Reference population and validation population do not agree on directionality of association.

### Heterogeneity of Association with Cancer Occurrence among Ethnic Groups

**Figure 2:**
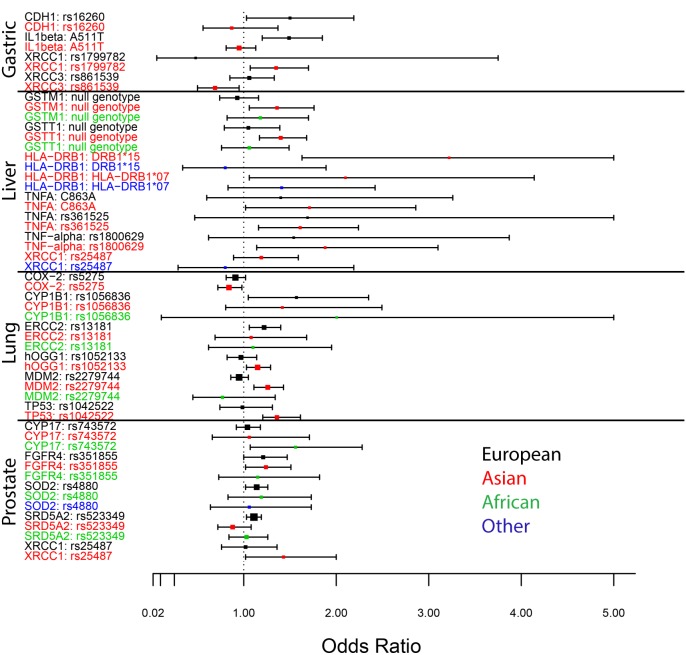
Forest plot of odds ratios. The results within liver, gastric, lung and prostate cancer are shown. OR’s from European populations are shown in black, Asian in red, African in green, and other groups in blue. Though considerably heterogeneity is apparent, the association with risk for a marker in one ethnic group appears to predict the direction of the association in the other ethnic groups, as supported by the test for heterogeneity. Similar plots for breast and colon cancer are given in Figure S1 and S2, respectively.

Though the most of the associations between genetic variants and cancer risk that were assessed in this study do not replicate between ethnic groups, this does not establish that there is no consistency of association for these variants across ethnic groups. Indeed, a survey of the odds ratios and confidence intervals in the studied loci suggests that the effect on cancer risk associated with the studied alleles may often be consistent across ethnic boundaries (Fig. 2, lung, gastric, liver, and prostate cancer; Fig. S1, breast cancer; Fig. S2, colorectal carcinoma). Though considerable variation is apparent among the ethnic groups, the direction of the association is often conserved. To more rigorously evaluate this, the differences of the odds ratio between ethnic groups was assessed using the Breslow-Day test with Tarone’s adjustment [30] to determine whether there was significant heterogeneity among ethnic groups. The Breslow-Day test assesses the homogeneity of the odds ratio across contingency tables and has an approximate chi-squared distribution. Loci were excluded if incomplete case and control numbers for each ethnic group were not reported. Only a minority of loci showed significant heterogeneity among ethnic groups (25%, 15/60 SNPs, Table 2). There were some differences between the cancer types, with two out of the four tested loci in gastric cancer showing significant heterogeneity, but the number of loci is too small to statistically determine if there is a meaningful difference in heterogeneity between the different cancers. Excluding gastric cancer, loci exhibiting significant heterogeneity were in the minority, ranging from 8% (colon cancer) to 40% (prostate cancer). If the analysis is restricted to only include data from populations where a significant result was found or the study was well powered, similar results are found, with 67% (28/42) of loci showing non-significant heterogeneity among ethnic groups (data not shown). As discoveries of significant risk associations in small populations could skew the results, the analysis was also performed excluding discovery populations whose number of total participants were less than the 10^th^ percentile of this entire study (N<548). The results were not appreciably changed, five SNPs were affected and the number of loci showing significant heterogeneity was 26%. Therefore, by this measure, association with cancer risk is broadly consistent across ethnic boundaries; a finding of an association with risk in one population predicts the direction of that risk association in another ethnic group. However, as our results in table 1 demonstrate, this does not mean that one should expect a significant association in one ethnic group to lead to a significant result in another ethnic group.

**Table 2:** Heterogeneity of OR among ethnic groups.

	total SNPs	?^2^ P value <0.05	fraction total
**breast**	32	9	0.28
**colon**	13	1	0.08
**gastric**	4	2	0.50
**lung**	6	1	0.17
**prostate**	5	2	0.40
**Total**	60	15	0.25

Tarone’s Test for was used to assess heterogeneity of the odds ratios between ethnic groups. The fraction of SNP’s showing significant variability is tabulated.

### Linkage Disequilibrium Analysis

For sites of variation with disagreement between ethnic groups (as defined by significant results predicting increased or decreased risk for the same allele, or significant results in one group but non-significant yet powered analysis in another group), linkage disequilibrium (LD) analysis was performed. LD patterns between ethnic groups within a region 50 kilobase on each side of the locus of interest were compared. Looking at cases which had at least 20 SNP pairs available for LD analysis within this region, 62% of loci showed significant differences in the r^2^ of SNPs compared with the tested variant between ethnic groups and 23% of loci showed significant disagreement between ethnic groups in odds ratio, as assessed by a z test on the difference in the odds ratios (Table 3). A linear mixed model for the agreement of these tests showed significant association (p = 0.013). This result suggests that the agreement in OR between different ethnic groups is associated with the comparative variation in the surrounding genome structure. This likely reflects that in conserved regions the link between the tested marker and actual risk allele remain tight.

**Table 3:** Relationship between linkage disequilibrium and cancer susceptibility.

Cancer	SNP	ethnicity comparison	LD Agreement	OR agreement
breast	rs1137101	African-Asian	0.66	0.23
breast	rs1137101	African-European	0.01	0.01
breast	rs1137101	Asian-European	0.03	0.00
breast	rs13181	African-European	0.00	0.07
breast	rs1799793	Asian-European	0.00	0.13
breast	rs1801133	Asian-European	0.00	0.03
breast	rs2273535	Asian-European	0.49	0.39
liver	rs1800629	Asian-European	0.01	0.37
lung	rs1056836	African-Asian	0.01	0.07
lung	rs1056836	African-European	0.00	0.13
lung	rs1056836	Asian-European	0.13	0.13
lung	rs13181	Asian-European	0.05	0.35
lung	rs5275	Asian-European	0.56	0.17

Agreement between odds ratios was compared with a z test of the difference; z = δOR/SE (δOR). LD agreement was assessed with a one-way permutation test based on Monte-Carlo resampling on the r^2^ values between the relevant SNP and all available SNPs within 50 kb on either side of the loci. Two sided P values are shown.

## Discussion

Our results demonstrate that ethnic background usually plays an important role in affecting the association between a putative risk marker and cancer risk. In a survey of studies encompassing 96 risk:variant associations (82 unique alleles) in six cancers assessing the association between cancer susceptibility and allelic variations, we found that a significant result in one ethnic group was usually not reproducible on other ethnicities in well-powered studies. This is consistent with other studies [23], [24], [35], though this is the first large review to focus on cancer risk associations. Whether clinical studies are to be expected to validate has been a subject of interest of late [36], and there are many reasons why a result may fail to replicate. One hypothesis we initially entertained was that insufficient case numbers for rare alleles would account for the majority of disparate results. However this hypothesis was not supported, limiting analysis to well-powered studies still saw that most associations between variants and cancer risk did not replicate in different ethnic groups. However, we also saw that most loci exhibited consistency in their association with risk, most loci did not have statistically significant heterogeneity in the OR’s among the studied ethnic groups. These results are not contradictory, but the distinction is important in understanding the complicated manners that ethnic variation can affect clinical studies. The test for heterogeneity suggests that the basic biologic effect of a site of genetic variation may often be shared across ethnic boundaries. On the other hand, the power analyses suggests that, despite this putatively shared biology, reproducing a result found in one ethnic group may be difficult to achieve in another group. Therefore, although a basic biological effect may be conserved, the tested alleles’ contributions to cancer risk appear to include factors intrinsically distinct between ethnic groups. These factors are likely to confound efforts to translate utility of a marker from one ethnic group to another unless adequately accommodated.

The cause of the different association for a marker among ethnic groups could be due to either the risk alleles being linked to the real causative allele with differing strengths between the groups, the allele acting in different manners across ethnic boundaries in how it affects cancer risk, or differing interactions between the risk allele with environmental or other genetic elements that vary among populations. We present evidence that genetic linkage appears to be a strong factor in explaining the differing association between marker and risk for many of the tested alleles, consistent with findings in other studies [23]. This does not mean that environmental and higher level genetic interactions do not contribute to inter-ethnic diversity. These results do suggest that, when trying to translate genetic association results from one ethnic group to another, validation within all ethnic groups of interest is vitally important and efforts to identify causal genetic loci and or closely linked loci will improve conservation across ethnic boundaries.

**Figure 3:**
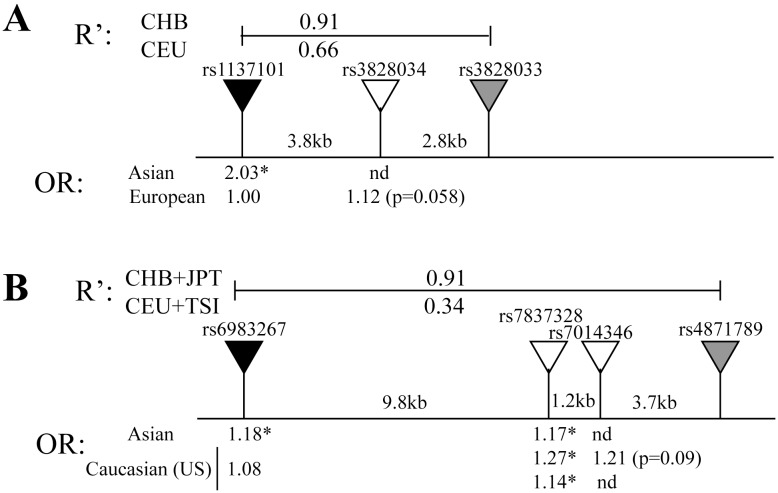
Using ethnic variation to nominate better candidate markers. Black triangles represent a SNP which exhibited significant association with risk in one population and non-significant association in a different ethnic group. Gray triangles are SNPs that are tightly linked to this marker in the population with a significant association but more loosely linked in the non-significant population. White triangles are SNPs very close to this new candidate region with a measured association with outcome. The OR’s shown below the black marker are from this study, those under the gray and white markers from the referenced studies. Significant results are marked with asterisks. A) rs1137101 failed to validate in a European breast cancer population, however the nearby rs3828034 has a higher OR that nears significance [46]. B) rs6983267 failed to replicate in studies of European (US) populations, however the nearby rs7837328 has a more consistent association [47]–[49]. The odds ratio for rs6983267 as reported in this study (Table S2) is based on the ancestral allele, which is also the rare allele in European populations, the odds ratio for the nearby SNPs were reported in relation to the most common allele, therefore for consistency we have also given the OR for rs6983267 in this figure in relation to the common allele.

The results reported here suggest that the linkage between commonly utilized or studied cancer risks markers at defined risk loci are often poorly linked to the actual risk alleles. Using this genetic diversity among populations may therefore allow better mapping of these true risk alleles. As LD structure has been demonstrated to vary among ethnic groups, studies assessing multiple ethnic groups can greatly aid these types of efforts [37]. Allelic variation in high LD with a marker linked to risk in a studied population serves as candidates of possible risk alleles to be assessed in the index ethnic group and yet untested populations. For example, fine-mapping in Asian, European, and African-Americans in a FGFR2 associated allele in breast cancer led to better definition of the risk region [38]. In this regard, the linkage differences among ethnic populations may be useful for the nomination of SNPs that are more closely linked to the true functional SNP. Therefore, SNPs in high LD to the tested risk marker in the ethnic group with the significant association, but more loosely linked in the group with a non-significant association may indicate regions where the true functional SNP resides. As an example, in figure 3 we show examples from breast (Fig. 3A) and colon (Fig. 3B) cancers. In each case, SNPs with a more consistent association with cancer across ethnic boundaries were found in regions nearby the initial tested markers. Continued fine mapping of variants, and increased reporting of all results from GWAS (not just the markers that meet the corrected significance levels required in the identification of novel markers) will greatly speed up the ability to use such information to identify risk markers that translate across ethnic boundaries.

As understanding of the genetic variation among disparate population groups is of clear importance in assessing cancer risk, the risks of using self-reported ethnic designations as surrogates for complete genetic information must be considered as a limitation of this study, and of any study that uses self-reported ethnicity. Potential problems in the use of ancestry identifiers (such as race and ethnicity) in medical studies have been addressed in several reports [39]–[43]. These ethnic labels are surrogates, with significant short-comings, for the shared genetic variation and shared genetic history that explain the differences in allele frequencies observed between population groups [44], [45]. However the factors that comprise self-reported ethnicity may encompass elements common to genetically distinct groups, such as shared cultural and historical experiences, beliefs and rituals, and other customs. While these elements may also be important in creating a complete risk model for an individual, distinguishing these different types of factors is important, and the use of self-reported ethnic labels may not contribute to their differentiation. Nonetheless, until fine scale mapping or sequencing of individuals becomes the norm in medical diagnostic and therapeutic decision making, the use of ethnic group labels appears necessary.

## Supporting Information

Figure S1 **Forest plot of odds ratios for breast cancer.** OR’s from European populations are shown in black, Asian in red, African in green, and other groups in blue.
(TIF)

Figure S2 **Forest plot of odds ratios for colon cancer.** OR’s from European populations are shown in black, Asian in red, African in green, and other groups in blue.
(TIF)

Figure S3 **Funnel plots for assessment of publication bias.** Plots are shown for each cancer, and within breast, each ethnic group. Egger’s regression test is used to assess the significance of deviation from symmetry; the P value for this test is shown. A) breast cancer, all populations; B) breast cancer, European populations; C) breast cancer, Asian populations; D) breast cancer, African populations; E) colon cancer, all populations; F) lung cancer, all populations; G) Gastric cancer, all populations; H) Liver cancer, all populations; I) Prostate cancer, all populations.
(TIF)

Table S1 **Variations in this study and their known prevalence in the studied ethnic categories.**
(DOCX)

Tables S2 **The association of the assessed variations with risk of breast cancer [51]–[91].**
(DOCX)

Tables S3 **The association of the assessed variations with risk of colon cancer [92]–[102].**
(DOCX)

Tables S4 **The association of the assessed variations with risk of liver cancer [103]–[106].**
(DOCX)

Tables S5 **The association of the assessed variations with risk of lung cancer [107]–[110].**
(DOCX)

Tables S6 **The association of the assessed variations with risk of gastric cancer [111]–[116].**
(DOCX)

Tables S7 **The association of the assessed variations with risk of prostate cancer [117]–[121].**
(DOCX)

Checklist S1 **PRISMA Checklist.**
(DOCX)
