# Supplementary figures and images for: Ethnic Background and Genetic Variation in the Evaluation of Cancer Risk: A Systematic Review
Source: PLoS One. 2014 Jun 5;9(6):e97522. doi: 10.1371/journal.pone.0097522 (PMC4046957; doi:10.1371/journal.pone.0097522)

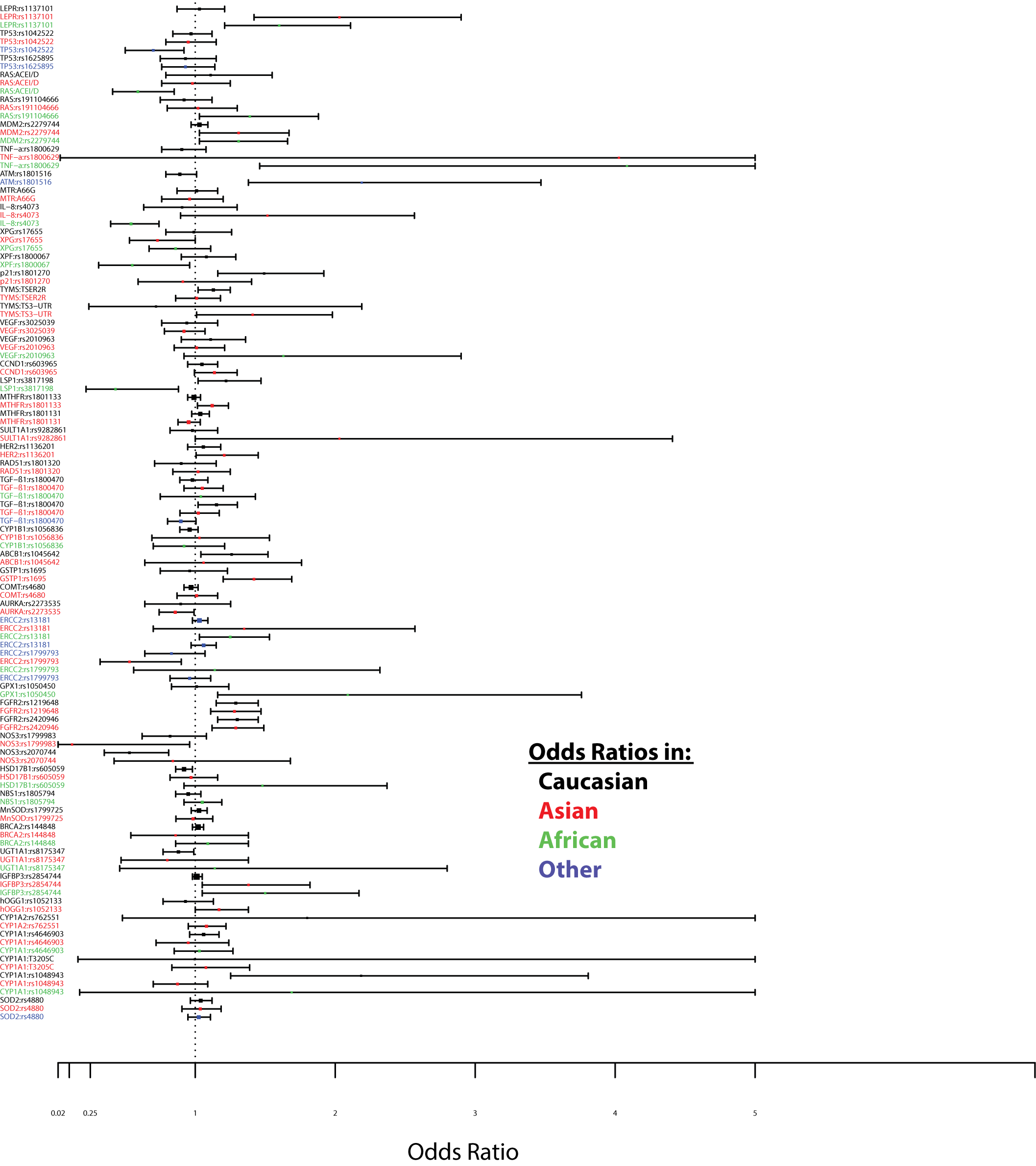

Supplement: Figure S1 — Forest plot of odds ratios for breast cancer. OR’s from European populations are shown in black, Asian in red, African in green, and other groups in blue. (TIF) [file pone.0097522.s001.tif]

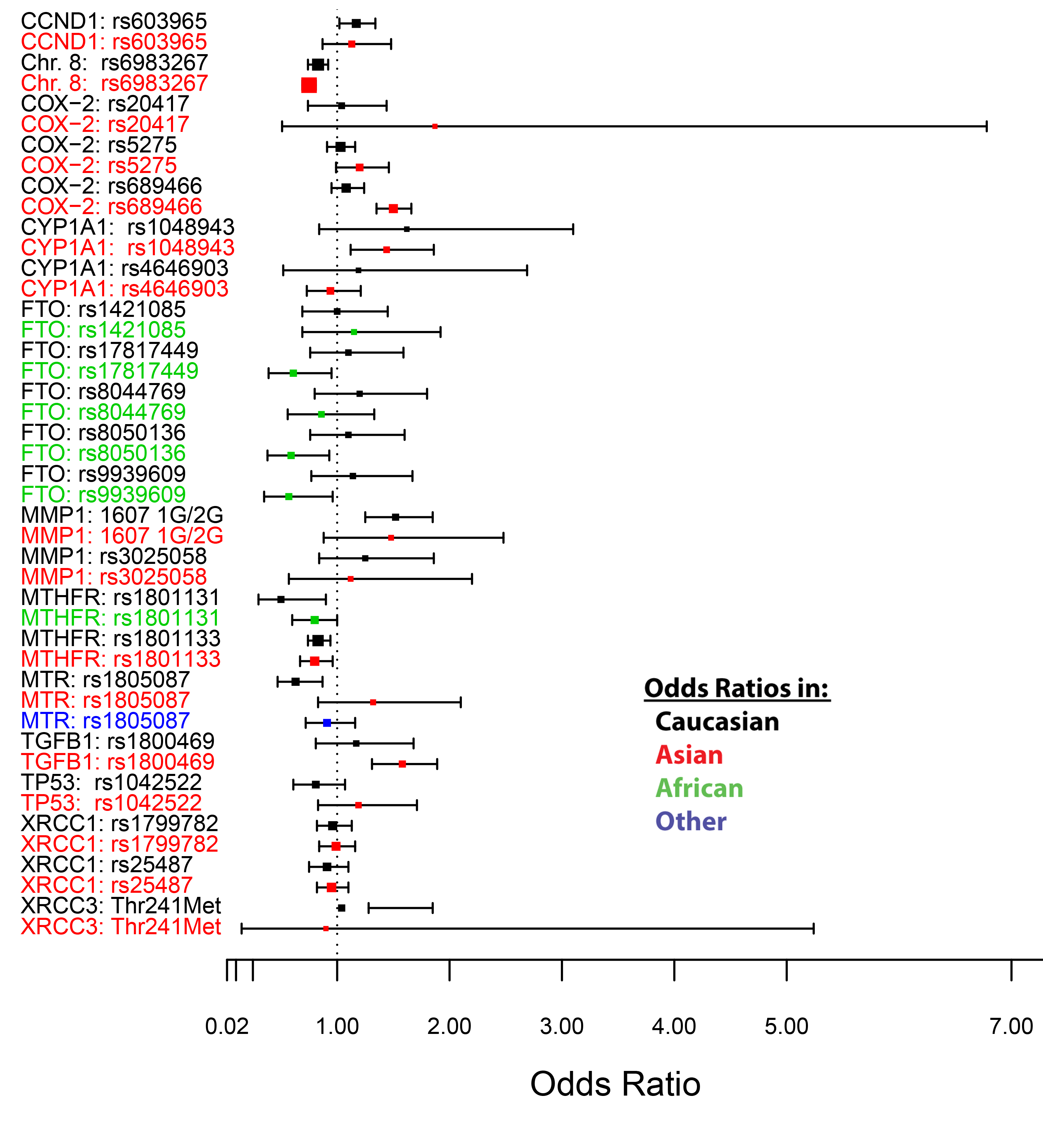

Supplement: Figure S2 — Forest plot of odds ratios for colon cancer. OR’s from European populations are shown in black, Asian in red, African in green, and other groups in blue. (TIF) [file pone.0097522.s002.tif]

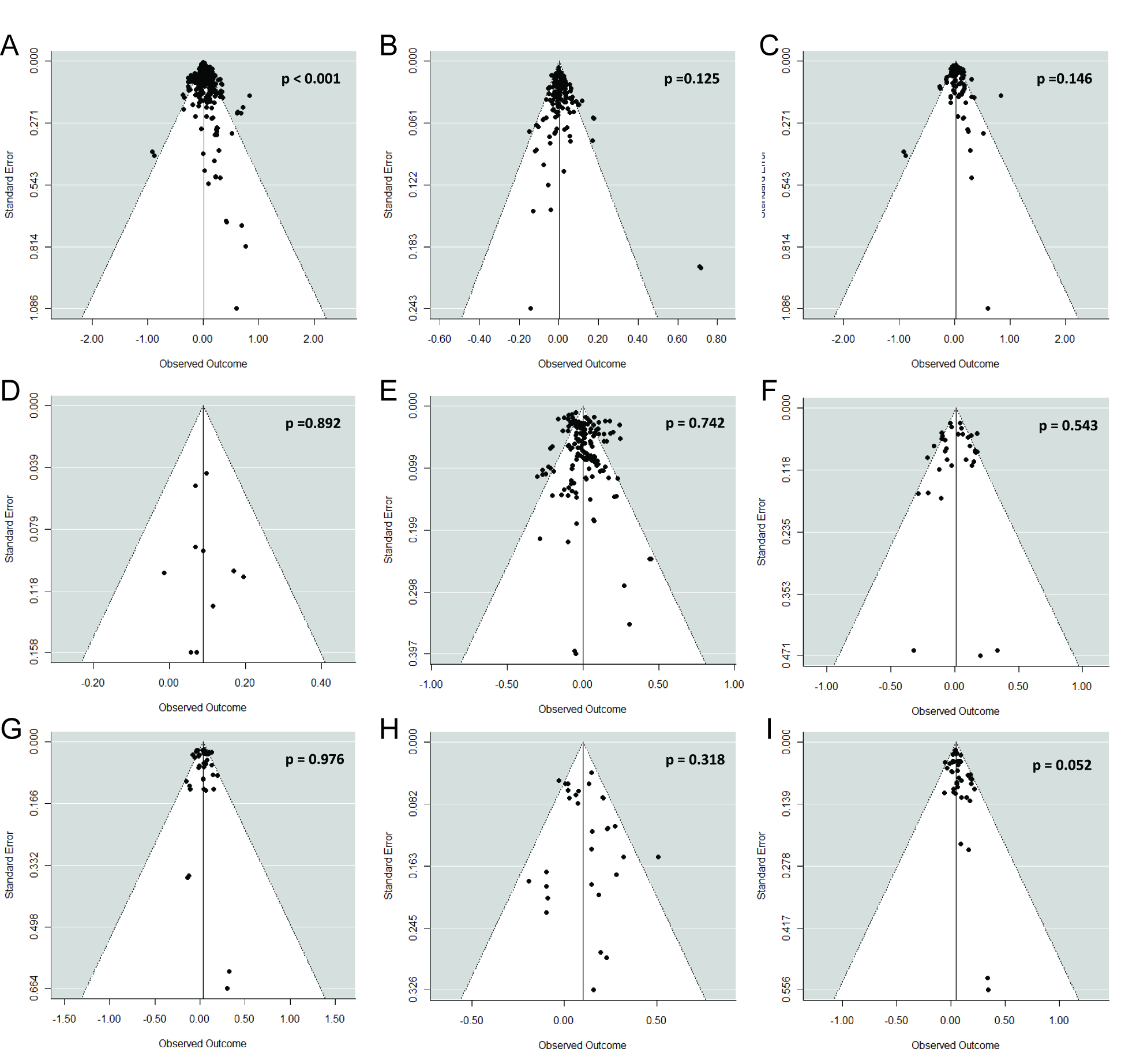

Supplement: Figure S3 — Funnel plots for assessment of publication bias. Plots are shown for each cancer, and within breast, each ethnic group. Egger’s regression test is used to assess the significance of deviation from symmetry; the P value for this test is shown. A) breast cancer, all populations; B) breast cancer, European populations; C) breast cancer, Asian populations; D) breast cancer, African populations; E) colon cancer, all populations; F) lung cancer, all populations; G) Gastric cancer, all populations; H) Liver cancer, all populations; I) Prostate cancer, all populations. (TIF) [file pone.0097522.s003.tif]
