# Supplementary material for: Ethnic Background and Genetic Variation in the Evaluation of Cancer Risk: A Systematic Review
Source: PLoS One. 2014 Jun 5;9(6):e97522. doi: 10.1371/journal.pone.0097522 (PMC4046957; doi:10.1371/journal.pone.0097522)
Supplement: Table S1 — Variations in this study and their known prevalence in the studied ethnic categories. (DOCX) [file pone.0097522.s004.docx]

Table S1 Variations in this study

|  |  |  |  |  | **Frequency of ancestral allele** | | | |  | | |
| --- | --- | --- | --- | --- | --- | --- | --- | --- | --- | --- | --- |
| **cancer** | **Gene** | **Variant** | **Alias** | **Ancestral allele** | **European** | **Han Chinese** | **Japanese** | **African** | **Source** |  |  |
| breast | ABCB1 | rs1045642 |  | A | 0.57 | 0.37 | 0.45 | 0.11 | 1 | |  |
| breast | ATM | rs1801516 |  | G | 0.81 | 0.99 | 1.00 | 1.00 | 1 | |  |
| breast | AURKA | rs2273535 | T91A | A | 0.82 | 0.37 | 0.38 | 0.82 | 1 | |  |
| breast | BRCA2 | rs144848 |  | T | 0.68 | 0.75 | 0.75 | 0.91 | 1 | |  |
| breast | CCND1 | rs603965 |  | G | 0.49 | 0.44 | 0.55 | 0.82 | 1 | |  |
| breast | COMT | rs4680 | Val158Met | G | 0.52 | 0.71 | 0.71 | 0.69 | 1 | |  |
| breast | CYP1A1 | rs1048943 | Ile462Val | A | 0.97 | 0.75 | 0.81 | 1.00 | 1 | |  |
| breast | CYP1A1 | rs4646903 |  | T | 0.90 | 0.63A | 0.63A | 0.86 | 2 | |  |
| breast | CYP1A1 | T3205C |  | T | not available | | | | 1 | |  |
| breast | CYP1A2 | rs762551 |  | C | 0.28 | 0.33 | 0.40 | 0.43 | 1 | |  |
| breast | CYP1B1 | rs1056836 | Val432Leu | G | 0.45 | 0.10 | 0.10 | 0.88 | 1 | |  |
| breast | ERCC2 | rs13181 |  | T | 0.67 | 0.91 | 0.94 | 0.82 | 1 | |  |
| breast | ERCC2 | rs1799793 |  | G | 0.70 | 0.93 | 0.90 | 0.93 | 1 | |  |
| breast | FGFR2 | rs1219648 |  | A | 0.54 | 0.61 | 0.66 | 0.57 | 1 | |  |
| breast | FGFR2 | rs2420946 |  | T | 0.47 | 0.41 | 0.34 | 0.58 | 1 | |  |
| breast | GPX1 | rs1050450 | Pro198Leu | C | not available | | | | 1 | |  |
| breast | GSTP1 | rs1695 | Ile105Val | A | 0.59 | 0.82 | 0.91 | 0.60 | 1 | |  |
| breast | HER2 | rs1136201 | Ile655Val | A | 0.74 | 0.78 | 0.86 | 1.00 | 1 | |  |
| breast | hOGG1 | rs1052133 |  | C | 0.79 | 0.50 | 0.48 | 0.86 | 1 | |  |
| breast | HSD17B1 | rs605059 | Ser312Gly | T | 0.57 | 0.42A | 0.42A | 0.39 | 2 | |  |
| breast | IGFBP3 | rs2854744 |  | C | 0.68 | 0.33A | 0.33A | 0.39 | 2 | |  |
| breast | IL-8 | rs4073 |  | A | 0.40 | 0.39 | 0.27 | 0.83 | 3 | |  |
| breast | LEPR | rs1137101 | Gln223Arg | A | 0.53 | 0.11 | 0.17 | 0.40 | 1 | |  |
| breast | LSP1 | rs3817198 |  | T | 0.67 | 0.89 | 0.89 | 0.91 | 1 | |  |
| breast | MDM2 | rs2279744 |  | T | 0.70 | 0.36A | 0.36A | 0.92 | 2 | |  |
| breast | MnSOD | rs1799725 |  | A | 0.55 | 0.87 | 0.90 | 0.62 | 1 | |  |
| breast | MTHFR | rs1801131 | A1298C | A | 0.66 | 0.81 | 0.81 | 0.88 | 1 | |  |
| breast | MTHFR | rs1801133 | C677T | C | 0.69 | 0.52 | 0.61 | 0.91 | 1 | |  |
| breast | MTR | A66G |  | A | not available | | | | 1 | |  |
| breast | NBS1 | rs1805794 | G8360C | C | 0.70 | 0.56 | 0.57 | 0.82 | 1 | |  |
| breast | NOS3 | rs1799983 |  | T | 0.35 | 0.11 | 0.07 | 0.06 | 1 | |  |
| breast | NOS3 | rs2070744 |  | C | 0.00 | 0.00 | 0.00 | 0.00 | 1 | |  |
| breast | p21 | rs1801270 |  | C | 0.96 | 0.55 | 0.60 | 0.69 | 1 | |  |
| breast | RAD51 | rs1801320 |  | G | 0.94 | 0.87 | 0.85 | 0.76 | 1 | |  |
| breast | RAS | rs4340 | ACE I/D | na | not available | | | | 1 | |  |
| breast | RAS | rs191104666 |  | T | not available | | | | 1 | |  |
| breast | SOD2 | rs4880 | Val16Ala | A | 0.55 | 0.87 | 0.90 | 0.62 | 1 | |  |
| breast | SULT1A1 | rs9282861 | Arg213His | G | not available | | | | 1 | |  |
| breast | TGF-β1 | rs1800470 | T29C | C | 0.00 | 0.00 | 0.00 | 0.00 | 1 | |  |
| breast | TNF-α | rs1800629 | G308A | G | 0.83 | 0.92 | 0.98 | 0.91 | 1 | |  |
| breast | TP53 | rs1042522 | Arg72Pro | C | 0.24 | 0.49 | 0.41 | 0.67 | 1 | |  |
| breast | TP53 | rs1625895 |  | A | 0.10 | 0.03 | 0.00 | 0.31 | 1 | |  |
| breast | TYMS | TS3'-UTR |  | na | not available | | | | 1 | |  |
| breast | TYMS | TSER 2R |  | na | not available | | | | 1 | |  |
| breast | UGT1A1 | rs8175347 | TA-repeat | na | not available | | | | 1 | |  |
| breast | VEGF | rs2010963 | G634C | G | 0.60 | 0.62A | 0.62A | 0.73 | 2 | |  |
| breast | VEGF | rs3025039 | C936T | C | 0.81 | 0.82 | 0.82 | 0.93 | 3 | |  |
| breast | XPF | rs1800067 |  | G | 0.95 | 1.00 | 1.00 | 1.00 | 1 | |  |
| breast | XPG | rs17655 |  | C | 0.72 | 0.56 | 0.48 | 0.46 | 1 | |  |
| colon | CCND1 | rs603965 | G870A | G | 0.49 | 0.44 | 0.55 | 0.82 | 1 | |  |
| colon | Chr. 8 | rs10505477 |  | T | 0.46 | 0.39 | 0.29 | 0.92 | 1 | |  |
| colon | Chr. 8 | rs6983267 |  | G | 0.49 | 0.39 | 0.29 | 0.98 | 1 | |  |
| colon | COX-2 | rs20417 | G1195A | C | 0.82 | 0.93 | 0.94 | 0.59 | 1 | |  |
| colon | COX-2 | rs5275 |  | A | 0.62 | 0.83 | 0.76 | 0.39 | 1 | |  |
| colon | COX-2 | rs689466 |  | A | 0.83 | 0.51 | 0.57 | 0.91 | 1 | |  |
| colon | CYP1A1 | rs1048943 |  | A | 0.97 | 0.76 | 0.79 | 1.00 | 1 | |  |
| colon | CYP1A1 | rs4646903 |  | T | 0.90 | 0.63A | 0.63A | 0.86 | 2 | |  |
| colon | FTO | rs1421085 |  | T | 0.54 | 0.86 | 0.81 | 0.93 | 1 | |  |
| colon | FTO | rs17817449 |  | T | 0.54 | 0.86 | 0.82 | 0.61 | 1 | |  |
| colon | FTO | rs8044769 |  | T | 0.44 | 0.39 | 0.32 | 0.17 | 1 | |  |
| colon | FTO | rs8050136 |  | C | 0.54 | 0.86 | 0.82 | 0.54 | 1 | |  |
| colon | FTO | rs9939609 |  | T | 0.54 | 0.85 | 0.81 | 0.49 | 1 | |  |
| colon | MMP1 | 1607 1G/2G |  | C | not available | | | | 1 | |  |
| colon | MMP1 | rs3025058 | 1171 5A>6A | T | not available | | | | 1 | |  |
| colon | MTHFR | rs1801131 | A1298C | A | 0.66 | 0.81 | 0.81 | 0.88 | 1 | |  |
| colon | MTHFR | rs1801133 | C677T | C | 0.69 | 0.52 | 0.61 | 0.91 | 1 | |  |
| colon | MTR | rs1805087 |  | A | 0.84 | 0.93 | 0.81 | 0.69 | 1 | |  |
| colon | TGFB1 | rs1800469 | C509T | A | 0.29 | 0.47 | 0.47 | 0.21 | 1 | |  |
| colon | TP53 | rs1042522 | Arg72Pro | C | 0.23 | 0.49 | 0.41 | 0.67 | 1 | |  |
| colon | XRCC1 | rs1799782 |  | C | 0.91 | 0.76 | 0.72 | 0.92 | 1 | |  |
| colon | XRCC1 | rs25487 |  | T | 0.37 | 0.25 | 0.27 | 0.11 | 1 | |  |
| colon | XRCC3 | Thr241Met |  | T | not available | | | | 1 | |  |
| gastric | CDH1 | rs16260 |  | C | 0.72 | 0.76 | 0.81 | 0.84 | 1 | |  |
| gastric | IL1beta | A511T |  | A | not available | | | | 1 | |  |
| gastric | XRCC1 | rs1799782 |  | C | 0.91 | 0.76 | 0.72 | 0.92 | 1 | |  |
| gastric | XRCC3 | rs861539 |  | C | 0.57 | 0.94 | 0.89 | 0.83 | 1 | |  |
| liver | GSTM1 | null genotype |  | na | not available | | | | 1 | |  |
| liver | GSTT1 | null genotype |  | na | not available | | | | 1 | |  |
| liver | HLA-DRB1 | DRB1*15 |  | na | not available | | | | 1 | |  |
| liver | HLA-DRB1 | HLA-DRB1*07 |  | na | not available | | | | 1 | |  |
| liver | TNFA | C863A |  | C | not available | | | | 1 | |  |
| liver | TNFA | rs361525 | G238A | G | 0.93 | 0.96 | 1.00 | 0.99 | 3 | |  |
| liver | TNF-α | rs1800629 | G308A | G | 0.83 | 0.92 | 0.98 | 0.91 | 1 | |  |
| lung | COX-2 | rs5275 |  | A | 0.62 | 0.83 | 0.76 | 0.39 | 1 | |  |
| lung | CYP1B1 | rs1056836 |  | G | 0.45 | 0.08 | 0.10 | 0.88 | 1 | |  |
| lung | ERCC2 | rs13181 |  | T | 0.67 | 0.90 | 0.92 | 0.81 | 1 | |  |
| lung | hOGG1 | rs1052133 |  | C | 0.79 | 0.50 | 0.48 | 0.86 | 1 | |  |
| lung | MDM2 | rs2279744 |  | T | 0.70 | 0.36A | 0.36A | 0.92 | 2 | |  |
| lung | TP53 | rs1042522 | Arg72Pro | C | 0.24 | 0.49 | 0.41 | 0.67 | 1 | |  |
| prostate | CYP17 | rs743572 |  | A | 0.62 | 0.41 | 0.54 | 0.71 | 1 | |  |
| prostate | FGFR4 | rs351855 | Gly388Arg | C | 0.72 | 0.54 | 0.58 | 0.89 | 1 | |  |
| prostate | SOD2 | rs4880 | Val16Ala | A | 0.55 | 0.87 | 0.90 | 0.62 | 1 | |  |
| prostate | SRD5A2 | rs523349 | Val89Leu | G | 0.19 | 0.48 | 0.36 | 0.23 | 1 | |  |
| prostate | XRCC1 | rs25487 |  | T | 0.37 | 0.25 | 0.27 | 0.11 | 1 | |  |

1. International HapMap Project[1]
2. 1000 Genomes low coverage panel[2]
3. SeattleSNPs genome panel (<http://pga.gs.washington.edu/>

*References*

1. (2003) The International HapMap Project. Nature 426: 789-796.

2. Genomes Project Consortium, Abecasis GR, Altshuler D, Auton A, Brooks LD, et al. (2010) A map of human genome variation from population-scale sequencing. Nature 467: 1061-1073.
