# Supplementary material for: Ethnic Background and Genetic Variation in the Evaluation of Cancer Risk: A Systematic Review
Source: PLoS One. 2014 Jun 5;9(6):e97522. doi: 10.1371/journal.pone.0097522 (PMC4046957; doi:10.1371/journal.pone.0097522)
Supplement: Tables S2 — The association of the assessed variations with risk of breast cancer [51]–[91]. (DOCX) [file pone.0097522.s005.docx]

Table S2 Associations with breast cancer

| **Gene** | **SNP** | **Model** | **Ethnicity** | **# of studies** | **# of cases** | **# of controls** | **Odd's Ratio** | **Power** | **Reference** |
| --- | --- | --- | --- | --- | --- | --- | --- | --- | --- |
| ABCB1 | rs1045642 | 'C vs R' OR 'Allele' | European | 3 | 449 | 523 | 1.26 (1.04-1.52) | NA | [50] |
|  |  |  | Asian | 4 | 303 | 245 | 1.06(0.64-1.76) | 0.14 |  |
|  |  | RR vs CC | European | 3 | 262 | 290 | 1.48(1.04-2.11) | NA |  |
|  |  |  | Asian | 4 | 144 | 118 | 1.14(0.38-3.42) | 0.25 |  |
|  |  | CC vs CR | European | 3 | 335 | 363 | 1.13(0.81-1.58) | NA |  |
|  |  |  | Asian | 4 | 231 | 184 | 1.19(0.59-2.41) | NA |  |
|  |  | Dominant | European | 3 | 449 | 523 | 0.79(0.59-1.06) | NA |  |
|  |  |  | Asian | 4 | 303 | 245 | 0.84(0.37-1.89) | NA |  |
|  |  | **Recessive** | **European** | **3** | **449** | **523** | **0.71(0.52-0.96)** | **NA** |  |
|  |  |  | **Asian** | **4** | **303** | **245** | **1.00(0.53-1.92)** | **0.1** |  |
| ATM | rs1801516 | RR vs CC | European | 6 | 1935 | 1456 | 0.77(0.58–1.03) | NA | [51] |
|  |  |  | Amerindian | 3 | 241 | 374 | 1.24(0.11–13.85) | NA |  |
|  |  | **CC vs CR** | **European** | **6** | **1935** | **1456** | **0.89(0.79–1.01)** | **1** |  |
|  |  |  | **Amerindian** | **3** | **241** | **374** | **2.19(1.38–3.47)** | **NA** |  |
|  |  | Dominant | European | 6 | 2580 | 1941 | 0.88(0.78–0.99) | NA |  |
|  |  |  | Amerindian | 3 | 321 | 499 | 2.15(1.37–3.38) | NA |  |
|  |  | Recessive | European | 6 | 2580 | 1941 | 0.84(0.65–1.09) | NA |  |
|  |  |  | Amerindian | 3 | 321 | 499 | 0.99(0.45–2.15) | NA |  |
| AURKA | rs2273535 | **CC vs CR** | **European** | **6** | **6534** | **9455** | **0.896 (0.640–1.253)** | **1** | [52] |
|  |  |  | **Asian** | **3** | **1235** | **2063** | **0.857 (0.742-0.991)** | **NA** |  |
|  |  | Dominant | European | 6 | 7250 | 10354 | 0.996 (0.971–1.021) | NA |  |
|  |  |  | Asian | 3 | 2329 | 3675 | 0.894 (0.768–1.041) | NA |  |
|  |  | Recessive | European | 6 | 7250 | 10354 | 0.963 (0.776–1.194) | NA |  |
|  |  |  | Asian | 3 | 2329 | 3675 | 0.816 (0.621–-1.071) | NA |  |
| BRCA2 | rs144848 | RR vs CC | European | 19 | 15673 | 15385 | 1.05 (0.97–1.13) | NA | [53] |
|  |  |  | Asian | 1 | 112 | 116 | 1.63 (0.38–7.02) | NA |  |
|  |  |  | African | 1 | 572 | 506 | 1.13 (0.52–2.44) | NA |  |
|  |  | CC vs CR | European | 19 | 15673 | 15385 | 1.01 (0.97–1.05) | NA |  |
|  |  |  | Asian | 1 | 112 | 116 | 0.82 (0.51–1.33) | NA |  |
|  |  |  | African | 1 | 572 | 506 | 1.08 (0.85–1.38) | NA |  |
|  |  | Dominant | European | 19 | 20897 | 20513 | 1.02 (0.98–1.06) | NA |  |
|  |  |  | Asian | 1 | 149 | 154 | 0.86 (0.54–1.38) | NA |  |
|  |  |  | African | 1 | 762 | 675 | 1.09 (0.86–1.38) | NA |  |
|  |  | **Recessive** | **European** | **19** | **20897** | **20513** | **1.05 (0.97–1.13)** | **NA** |  |
|  |  |  | **Asian** | **1** | **149** | **154** | **1.75 (0.41–7.45)** | **NA** |  |
|  |  |  | **African** | **1** | **762** | **675** | **1.11 (0.52–2.39)** | **NA** |  |
| CCND1 | rs603965 | RR vs CC | European | 9 | 5478 | 6112 | 1.093 (0.959–1.246) | NA | [54] |
|  |  |  | Chinese | 4 | 1955 | 2267 | 1.144 (0.984–1.329) | NA |  |
|  |  | CC vs CR | European | 9 | 5478 | 6112 | 1.025 (0.918–1.144) | NA |  |
|  |  |  | Chinese | 4 | 1955 | 2267 | 1.054 (0.800–1.389) | NA |  |
|  |  | **Dominant** | **European** | **9** | **7304** | **8149** | **1.048 (0.946–1.160)** | **NA** |  |
|  |  |  | **Chinese** | **4** | **2607** | **3022** | **1.137 (0.994–1.299)** | **NA** |  |
|  |  | Recessive | European | 9 | 7304 | 8149 | 1.063 (0.984–1.149) | NA |  |
|  |  |  | Chinese | 4 | 2607 | 3022 | 1.084 (0.908–1.294) | NA |  |
| COMT | rs4680 | RR vs CC | European | 22 | 8152 | 11213 | 0.96 (0.87–1.06) | NA | [55] |
|  |  |  | Asian | 13 | 2442 | 3005 | 0.98 (0.73–1.33) | NA |  |
|  |  | CC vs CR | European | 22 | 11989 | 16092 | 0.98 (0.93–1.04) | NA |  |
|  |  |  | Asian | 13 | 3719 | 4559 | 1.01 (0.86–1.19) | NA |  |
|  |  | Dominant | European | 22 | 16276 | 22176 | 0.97 (0.92–1.02) | NA |  |
|  |  |  | Asian | 13 | 4021 | 4932 | 1.01 (0.87–1.16) | NA |  |
|  |  | **Recessive** | **European** | **22** | **16276** | **22176** | **0.97 (0.89–1.05)** | **NA** |  |
|  |  |  | **Asian** | **13** | **4021** | **4932** | **0.98 (0.73–1.32)** | **NA** |  |
| CYP1A1 | rs1048943 | RR vs CC | European | 18 | 6765 | 12347 | 2.185 ( 1.253–3.808) | NA | [56] |
|  |  |  | Chinese | 8 | 2161 | 2594 | 0.873 (0.700–1.089) | 1 |  |
|  |  |  | African | 3 | 267 | 344 | 1.689 (0.174–16.378) | 0.9 |  |
|  |  | CCvs CR | European | 18 | 6765 | 12347 | 1.062 (0.852–1.323) | NA |  |
|  |  |  | Chinese | 8 | 2161 | 2594 | 0.997 (0.892–1.115) | NA |  |
|  |  |  | African | 3 | 267 | 344 | 1.322 (0.637–2.743) | NA |  |
|  |  | Dominant | European | 18 | 9020 | 16462 | 1.115 (0.911–1.366) | NA |  |
|  |  |  | Chinese | 8 | 2881 | 3458 | .952 (0.860–1.055) | NA |  |
|  |  |  | African | 3 | 356 | 459 | 1.322 (0.637–2.743) | NA |  |
|  |  | **Recessive** | **European** | **18** | **9020** | **16462** | **2.076 ( 1.193– 3.614)** | **NA** |  |
|  |  |  | **Chinese** | **8** | **2881** | **3458** | **0.876 (0.708–1.086)** | **0.97** |  |
|  |  |  | **African** | **3** | **356** | **459** | **1.658 (0.171–16.068)** | **0.08** |  |
| CYP1A1 | rs4646903 | RR vs CC | European | 6 | 3585 | 6770 | 1.36 (0.88-2.11) | NA | [57] |
|  |  |  | Asian | 10 | 2053 | 2115 | 0.85 (0.56-1.27) | NA |  |
|  |  |  | African | 5 | 506 | 557 | 0.88 (0.47-1.65) | NA |  |
|  |  | CR vs RR | European | 6 | 726 | 1455 | 1.37 (0.87-2.14) | NA |  |
|  |  |  | Asian | 10 | 2302 | 2255 | 0.88 (0.66-1.17) | NA |  |
|  |  |  | African | 5 | 305 | 358 | 0.90 (0.59-1.39) | NA |  |
|  |  | Dominant | European | 6 | 4281 | 8152 | 1.06 (0.96-1.17) | NA |  |
|  |  |  | Asian | 10 | 3748 | 3792 | 0.95 (0.72-1.24) | NA |  |
|  |  |  | African | 5 | 763 | 864 | 1.03 (0.85-1.27) | NA |  |
|  |  | **Recessive** | **European** | **6** | **4281** | **8152** | **1.36 (0.88-2.11)** | **NA** |  |
|  |  |  | **Asian** | **10** | **3748** | **3792** | **0.85 (0.61-1.20)** | **NA** |  |
|  |  |  | **African** | **5** | **763** | **864** | **0.93 (0.62-1.40)** | **NA** |  |
| CYP1A1 | T3205C | RR vs CC | European | 3 | 455 | 578 | 1.052 (0.129–8.590) | NA | [56] |
|  |  |  | Asian | 5 | 578 | 654 | 0.917 (0.241–3.486) | NA |  |
|  |  | CC vs CR | European | 3 | 455 | 578 | 1.580 (0.222–11.267) | NA |  |
|  |  |  | Asian | 5 | 578 | 654 | 1.110 (0.856–1.440) | NA |  |
|  |  | Dominant | European | 3 | 607 | 770 | 0.996 (0.162–6.140) | NA |  |
|  |  |  | Asian | 5 | 771 | 872 | 1.076 (0.833–1.389) | NA |  |
|  |  | **Recessive** | **European** | **3** | **607** | **770** | **1.052 (0.129–8.589)** | **NA** |  |
|  |  |  | **Asian** | **5** | **771** | **872** | **0.911 (0.240–3.461)** | **NA** |  |
| CYP1A2 | rs762551 | RR vs CC | European | 2 | 1962 | 3347 | 5.87 (0.15-223.37) | NA | [58] |
|  |  |  | Asian | 3 | 1135 | 351 | 1.32 (0.77-2.26) | NA |  |
|  |  | CC vs CR | European | 2 | 2951 | 5213 | 1.43 (0.57-3.58) | NA |  |
|  |  |  | Asian | 3 | 1846 | 1052 | 1.06 (0.93-1.21) | NA |  |
|  |  | Dominant | European | 2 | 3240 | 5665 | 1.80 (0.48-6.77) | NA |  |
|  |  |  | Asian | 3 | 2117 | 1316 | 1.08 (0.95-1.22) | NA |  |
|  |  | **Recessive** | **European** | **2** | **3240** | **5665** | **4.92 (0.19-125.6)** | **NA** |  |
|  |  |  | **Asian** | **3** | **2117** | **1316** | **1.27 (0.75- 2.16)** | **NA** |  |
| CYP1B1 | rs1056836 | RR vs CC | European | 10 | 5807 | 6818 | 0.95(0.88-1.03) | NA | [59] |
|  |  |  | Asian | 5 | 2172 | 2170 | 1.00(0.67-1.50) | NA |  |
|  |  |  | African | 3 | 317 | 339 | 1.65(0.88-3.11) | NA |  |
|  |  | CR vs RR | European | 10 | 9214 | 10677 | 1.05(0.93-1.17) | NA |  |
|  |  |  | Asian | 5 | 2675 | 2733 | 0.91(0.80-1.03) | NA |  |
|  |  |  | African | 3 | 169 | 163 | 1.75(0.92-3.35) | NA |  |
|  |  | Dominant | European | 10 | 11445 | 13255 | 0.96(0.89-1.02) | NA |  |
|  |  |  | Asian | 5 | 2724 | 2783 | 1.03(0.69-1.53) | NA |  |
|  |  |  | African | 3 | 469 | 474 | 0.92(0.70-1.21) | NA |  |
|  |  | **Recessive** | **European** | **10** | **11445** | **13255** | **1.03(0.93-1.14)** | **NA** |  |
|  |  |  | **Asian** | **5** | **2724** | **2783** | **0.91(0.81-1.04)** | **NA** |  |
|  |  |  | **African** | **3** | **469** | **474** | **1.66 (0.89-3.09)** | **NA** |  |
| ERCC2 | rs13181 | RR vs CC | All white | 25 | 9200 | 9684 | 1.05 (0.97– 1.13) | NA | [60] |
|  |  |  | European | 13 | 5231 | 5594 | 1.04 (0.94 – 1.15) | NA |  |
|  |  |  | Asian | 5 | 1100 | 1267 | 1.60 (0.70– 3.66) | NA |  |
|  |  |  | White (N. America) | 11 | 3905 | 4003 | 1.06 (0.94 – 1.19) | NA |  |
|  |  |  | African American | 2 | 610 | 563 | 1.23 (0.81– 1.87) | NA |  |
|  |  | **Dominant** | **All white** | **25** | **12266** | **12912** | **1.03 (0.98 – 1.09)** | **1** |  |
|  |  |  | **European** | **13** | **6974** | **7458** | **1.02 (0.95 – 1.09)** | **1** |  |
|  |  |  | **Asian** | **5** | **1466** | **1689** | **1.35 (0.70 – 2.57)** | **0.63** |  |
|  |  |  | **White (N. American)** | **11** | **5206** | **5337** | **1.06 (0.97 – 1.15)** | **1** |  |
|  |  |  | **African American** | **2** | **813** | **751** | **1.25 (1.03–1.53)** | **NA** |  |
|  |  | Recessive | All white | 25 | 12266 | 12912 | 1.04 (0.95 – 1.13) | NA |  |
|  |  |  | European | 13 | 6974 | 7458 | 1.08 (0.93 – 1.25) | NA |  |
|  |  |  | Asian | 5 | 1466 | 1689 | 1.44 (0.80 – 2.61) | NA |  |
|  |  |  | White (N. American) | 11 | 5206 | 5337 | 0.99 (0.90 – 1.10) | NA |  |
|  |  |  | African American | 2 | 813 | 751 | 1.17 (0.78 – 1.77) | NA |  |
| ERCC2 | rs1799793 | RR vs CC | All white | 20 | 11023 | 9377 | 0.95 (0.83–1.08) | NA | [60] |
|  |  |  | European | 12 | 7554 | 6363 | 0.92 (0.77–1.11) | NA |  |
|  |  |  | Asian | 2 | 561 | 562 | 0.55 (0.32– 0.96) | NA |  |
|  |  |  | White (N. Americans) | 7 | 2379 | 2419 | 1.00 (0.85–1.18) | NA |  |
|  |  |  | African American | 2 | 607 | 562 | 1.18 (0.58–2.41) | NA |  |
|  |  | Dominant | All white | 20 | 14697 | 12502 | 1.13 (0.90–1.42) | NA |  |
|  |  |  | European | 12 | 10072 | 8484 | 1.14 (0.80–1.64) | NA |  |
|  |  |  | Asian | 2 | 748 | 755 | 0.96 (0.73–1.25) | NA |  |
|  |  |  | White (N. Americans) | 7 | 3172 | 3225 | 1.08 (0.98–1.19) | NA |  |
|  |  |  | African American | 2 | 809 | 749 | 1.17 (0.93–1.47) | NA |  |
|  |  | **Recessive** | **All white** | **20** | **14697** | **12502** | **0.83 (0.64–1.07)** | **1** |  |
|  |  |  | **European** | **12** | **10072** | **8484** | **0.77 (0.52–1.13)** | **1** |  |
|  |  |  | **Asian** | **2** | **748** | **755** | **0.53 (0.32–0.90)** | **NA** |  |
|  |  |  | **White (N. Americans)** | **7** | **3172** | **3225** | **0.96 (0.82–1.11)** | **0.99** |  |
|  |  |  | **African American** | **2** | **809** | **749** | **1.14 (0.56–2.32)** | **0.1** |  |
| FGFR2 | rs1219648 | RR vs CC | European | 3 | 1360 | 1366 | 1.63(1.21–2.19) | NA | [61] |
|  |  |  | Asian | 3 | 740 | 1038 | 1.46(1.18–1.80) | NA |  |
|  |  | CC vs CR | European | 3 | 2073 | 2216 | 1.19(1.05–1.34) | NA |  |
|  |  |  | Asian | 3 | 1240 | 1725 | 1.23(1.06–1.42) | NA |  |
|  |  | Dominant | European | 3 | 2619 | 2619 | 1.29(1.15–1.45) | NA |  |
|  |  |  | Asian | 3 | 1484 | 1974 | 1.28(1.11–1.47) | NA |  |
|  |  | **Recessive** | **European** | **3** | **2619** | **2619** | **1.46(1.17–1.83)** | **NA** |  |
|  |  |  | **Asian** | **3** | **1484** | **1974** | **1.30(1.07–1.58)** | **NA** |  |
| FGFR2 | rs2420946 | RR vs CC | European | 3 | 1365 | 1378 | 1.63(1.23–2.16) | NA | [61] |
|  |  |  | Asian | 3 | 728 | 1041 | 1.39(1.12–1.73) | NA |  |
|  |  | CC vs CR | European | 3 | 2076 | 2220 | 1.20(1.06–1.35) | NA |  |
|  |  |  | Asian | 3 | 1250 | 1721 | 1.25(1.08–1.45) | NA |  |
|  |  | Dominant | European | 3 | 2617 | 2620 | 1.30(1.16–1.45) | NA |  |
|  |  |  | Asian | 3 | 1473 | 1962 | 1.29(1.12–1.49) | NA |  |
|  |  | **Recessive** | **European** | **3** | **2617** | **2620** | **1.45(1.25–1.67)** | **NA** |  |
|  |  |  | **Asian** | **3** | **1473** | **1962** | **1.23(1.01–1.50)** | **NA** |  |
| GPX1 | rs1050450 | RR vs CC | European | 3 | 1149 | 1384 | 1.03 (0.84–1.27) | 1 | [62] |
|  |  |  | African | 1 | 54 | 308 | 1.91(1.02–3.58) | NA |  |
|  |  | Dominant | European | 3 | 2003 | 2378 | 1.06(0.86–1.30) | NA |  |
|  |  |  | African | 1 | 79 | 517 | 1.06(0.86–1.30) | NA |  |
|  |  | **Recessive** | **European** | **3** | **2003** | **2378** | **1.01 (0.83–1.24)** | **0.92** |  |
|  |  |  | **African** | **1** | **79** | **517** | **2.09 (1.16–3.76)** | **NA** |  |
| GSTP1 | rs1695 | RR vs CC | European | 8 | 4850 | 5739 | 0.96 (0.70–1.31) | 1 | [63] |
|  |  |  | Asian | 19 | 4111 | 4769 | 1.27 (1.02–1.83) | NA |  |
|  |  | CC vs CR | European | 8 | 7598 | 9372 | 0.98 (0.81–1.18) | NA |  |
|  |  |  | Asian | 19 | 5883 | 6838 | 1.07 (0.99–1.15) | NA |  |
|  |  | Dominant | European | 8 | 8497 | 10442 | 0.99 (0.81–1.20) | NA |  |
|  |  |  | Asian | 19 | 6209 | 7116 | 1.11 (0.98–1.26) | NA |  |
|  |  | **Recessive** | **European** | **8** | **8497** | **10442** | **0.96 (0.75–1.23)** | **0.95** |  |
|  |  |  | **Asian** | **19** | **6209** | **7116** | **1.42 (1.20–1.69)** | **NA** |  |
| HER2 | rs1136201 | RR vs CC | European | 13 | 3891 | 4743 | 1.046(0.852–1.283) | NA | [64] |
|  |  |  | Asian | 6 | 1247 | 1064 | 1.479(0.480–4.553) | NA |  |
|  |  | **CC vs CR** | **European** | **13** | **5642** | **6849** | **1.058(0.946–1.182)** | **1** |  |
|  |  |  | **Asian** | **6** | **1578** | **1307** | **1.207(1.006–1.450)** | **NA** |  |
|  |  | Dominant | European | 13 | 5948 | 7243 | 1.076(0.952–1.215) | NA |  |
|  |  |  | Asian | 6 | 1610 | 1325 | 1.218(0.993–1.494) | NA |  |
|  |  | Recessive | European | 13 | 5948 | 7243 | 0.992(0.847–1.161) | NA |  |
|  |  |  | Asian | 6 | 1610 | 1325 | 1.425(0.468–4.337) | NA |  |
| hOGG1 | rs1052133 | **RR vs CC** | **European** | **7** | **3247** | **3100** | **0.93 (0.77–1.13)** | **0.88** | [65] |
|  |  |  | **Asian** | **5** | **1781** | **1944** | **1.17 (1.00–1.38)** | **NA** |  |
|  |  | CC vs CR | European | 7 | 3247 | 3100 | 0.96 (0.88–1.05) | NA |  |
|  |  |  | Asian | 5 | 1781 | 1944 | 1.05 (0.91–1.22) | NA |  |
|  |  | Dominant | European | 7 | 4329 | 4133 | 0.96 (0.88–1.05) | NA |  |
|  |  |  | Asian | 5 | 2375 | 2592 | 1.10 (0.96–1.27) | NA |  |
|  |  | Recessive | European | 7 | 4329 | 4133 | 0.98 (0.82–1.18) | NA |  |
|  |  |  | Asian | 5 | 2375 | 2592 | 1.11 (0.99–1.25) | NA |  |
| HSD17B1 | rs605059 | RR vs CC | European | 4 | 3797 | 3881 | 0.91 (0.83-1.00) | NA | [66] |
|  |  |  | Asian | 3 | 684 | 1036 | 0.93 (0.76-1.14) | NA |  |
|  |  |  | African American | 1 | 82 | 103 | 1.30 (0.73-2.33) | NA |  |
|  |  | CC vs CR | European | 4 | 5832 | 6062 | 0.92 (0.85-0.99) | NA |  |
|  |  |  | Asian | 3 | 978 | 1391 | 0.99 (0.82-1.19) | 0.13 |  |
|  |  |  | African American | 1 | 135 | 143 | 1.57 (0.96-2.59) | 0.06 |  |
|  |  | **Dominant** | **European** | **4** | **7325** | **7671** | **0.92 (0.86-0.98)** | **NA** |  |
|  |  |  | **Asian** | **3** | **1352** | **2005** | **0.97 (0.82-1.16)** | **0.16** |  |
|  |  |  | **African American** | **1** | **177** | **189** | **1.48 (0.92-2.37)** | **0.07** |  |
|  |  | Recessive | European | 4 | 7325 | 7671 | 0.96 (0.89-1.04) | NA |  |
|  |  |  | Asian | 3 | 1352 | 2005 | 0.96 (0.82-1.12) | NA |  |
|  |  |  | African American | 1 | 177 | 189 | 0.97 (0.60-1.56) | NA |  |
| IGFBP3 | rs2854744 | RR vs CC | European | 20 | 22051 | 30197 | 1.05 (1.01-1.10) | NA | [67] |
|  |  |  | Asian | 3 | 1428 | 1463 | 1.35 (1.02-1.78) | NA |  |
|  |  |  | African | 1 | 213 | 314 | 1.27 (0.84-1.93) | 0.36 |  |
|  |  | CC vs CR | European | 20 | 22051 | 30197 | 1.04 (1.00-1.08) | 0.08 |  |
|  |  |  | Asian | 3 | 1428 | 1463 | 0.93 (0.82-1.07) | NA |  |
|  |  |  | African | 1 | 213 | 314 | 0.75 (0.53-1.06) | 0.06 |  |
|  |  | Dominant | European | 20 | 29401 | 40262 | 1.04 (1.00-1.08) | NA |  |
|  |  |  | Asian | 3 | 1904 | 1951 | 0.98 (0.86-1.11) | 0.09 |  |
|  |  |  | African | 1 | 284 | 418 | 0.89 (0.65-1.23) | 0.06 |  |
|  |  | **Recessive** | **European** | **20** | **29401** | **40262** | **1.01 (0.98-1.05)** | **0.96** |  |
|  |  |  | **Asian** | **3** | **1904** | **1951** | **1.38 (1.05-1.82)** | **NA** |  |
|  |  |  | **African** | **1** | **284** | **418** | **1.50 (1.05-2.17)** | **NA** |  |
| IL-8 | rs4073 | RR vs CC | European | 2 | 360 | 447 | 0.906(0.632–1.299) | 0.99 | [68] |
|  |  |  | Asian | 2 | 512 | 660 | 1.516(0.895–2.566) | 1 |  |
|  |  |  | African | 2 | 538 | 403 | 0.541(0.396–0.741) | NA |  |
|  |  | CC vs CR | European | 2 | 360 | 447 | 0.971 (0.720–1.309) | 0.64 |  |
|  |  |  | Asian | 2 | 512 | 660 | 1.444 (1.092–1.908) | NA |  |
|  |  |  | African | 2 | 538 | 403 | 0.867 (0.659–1.141) | 0.11 |  |
|  |  | **Dominant** | **European** | **2** | **480** | **596** | **1.059(0.659–1.703)** | **0.76** |  |
|  |  |  | **Asian** | **2** | **683** | **880** | **1.435(1.107–1.861)** | **NA** |  |
|  |  |  | **African** | **2** | **717** | **537** | **0.737(0.570–0.953)** | **NA** |  |
|  |  | Recessive | European | 2 | 480 | 596 | 0.884(0.515–1.518) | 0.3 |  |
|  |  |  | Asian | 2 | 683 | 880 | 1.194(0.614–2.321) | 0.4 |  |
|  |  |  | African | 2 | 717 | 537 | 0.594(0.459–0.768) | NA |  |
| LEPR | rs1137101 | RR vs CC | European | 4 | 1791 | 2033 | 1.00 (0.83-1.20) | 1 | [69] |
|  |  |  | Asian | 2 | 245 | 325 | 6.79 (3.42-13.47) | NA |  |
|  |  |  | African | 2 | 2724 | 2735 | 1.86 (1.28-2.71) | NA |  |
|  |  | CC vs CR | European | 4 | 2816 | 3268 | 1.05 (0.88-1.25) | 0.99 |  |
|  |  |  | Asian | 2 | 385 | 487 | 1.30 (0.85-1.97) | 0.64 |  |
|  |  |  | African | 2 | 3199 | 3171 | 1.48 (1.10-1.99) | NA |  |
|  |  | **Dominant** | **European** | **4** | **3305** | **3737** | **1.03 (0.87-1.21)** | **1** |  |
|  |  |  | **Asian** | **2** | **408** | **514** | **2.03 (1.42-2.90)** | **NA** |  |
|  |  |  | **African** | **2** | **3241** | **3210** | **1.60 (1.21-2.11)** | **NA** |  |
|  |  | Recessive | European | 4 | 3305 | 3737 | 0.95 (0.69-1.31) | 0.36 |  |
|  |  |  | Asian | 2 | 408 | 514 | 2.02 (0.20-21.04) | 0.1 |  |
|  |  |  | African | 2 | 3241 | 3210 | 1.48 (1.07-2.05) | NA |  |
| LSP1 | rs3817198 | 'C vs R' OR 'Allele' | European | 4 | 2970 | 2394 | 1.01 (0.94–1.09) | NA | [70] |
|  |  |  | African | 1 | 742 | 658 | 0.98 (0.81–1.20) | NA |  |
|  |  | RR vs CC | European | 4 | 1671 | 1354 | 1.25 (1.03–1.52) | NA |  |
|  |  |  | African | 1 | 518 | 483 | 0.45 (0.22-0.92) | NA |  |
|  |  | CC vs CR | European | 4 | 2639 | 2178 | 1.05 (0.94–1.18) | NA |  |
|  |  |  | African | 1 | 730 | 634 | 1.16 (0.92–1.47) | NA |  |
|  |  | Dominant | European | 4 | 2970 | 2394 | 1.09 (0.98–1.22) | NA |  |
|  |  |  | African | 1 | 742 | 658 | 1.08 (0.86–1.35) | NA |  |
|  |  | **Recessive** | **European** | **4** | **2970** | **2394** | **1.22 (1.02–1.47)** | **NA** |  |
|  |  |  | **African** | **1** | **742** | **658** | **0.43 (0.22–0.88)** | **NA** |  |
| MDM2 | rs2279744 | RR vs CC | European | 19 | 6837 | 6513 | 1.01 (0.89–1.14) | NA | [71] |
|  |  |  | Asian | 5 | 468 | 490 | 1.45 (0.83–2.54) | NA |  |
|  |  |  | African | 2 | 735 | 713 | 0.75 (0.39–1.47) | NA |  |
|  |  | **CC vs CR** | **European** | **19** | **10947** | **10159** | **1.03 (0.97–1.09)** | **1** |  |
|  |  |  | **Asian** | **5** | **731** | **738** | **1.31 (1.03– 1.67)** | **NA** |  |
|  |  |  | **African** | **2** | **916** | **838** | **1.31 (1.03– 1.66)** | **NA** |  |
|  |  | Dominant | European | 19 | 12770 | 11888 | 1.01 (0.96–1.07) | NA |  |
|  |  |  | Asian | 5 | 996 | 975 | 1.45 (0.92–2.26) | NA |  |
|  |  |  | African | 2 | 932 | 858 | 1.24 (0.99–1.56) | NA |  |
|  |  | Recessive | European | 19 | 12770 | 11888 | 1.01 (0.88–1.16) | NA |  |
|  |  |  | Asian | 5 | 996 | 975 | 0.99 (0.79–1.24) | NA |  |
|  |  |  | African | 2 | 932 | 858 | 0.72 (0.37–1.40) | NA |  |
| MnSOD | rs1799725 | RR vs CC | European | 28 | 17408 | 22073 | 1.013(0.939-1.092) | NA | [72] |
|  |  |  | Asian | 3 | 1539 | 1739 | 1.028(0.708-1.492) | NA |  |
|  |  | CC vs CR | European | 28 | 17408 | 22073 | 1.036(0.992-1.081) | NA |  |
|  |  |  | Asian | 3 | 1539 | 1739 | 0.978(0.852-1.122) | NA |  |
|  |  | Dominant | European | 28 | 23210 | 29430 | 1.027(0.971-1.085) | NA |  |
|  |  |  | Asian | 3 | 2052 | 2319 | 0.985(0.862-1.125) | NA |  |
|  |  | **Recessive** | **European** | **28** | **23210** | **29430** | **0.986(0.931-1.045)** | **NA** |  |
|  |  |  | **Asian** | **3** | **2052** | **2319** | **1.032(0.713-1.494)** | **NA** |  |
| MTHFR | rs1801131 | 'C vs R' OR 'Allele' | European | 7 | 4446 | 5315 | 1.036(0.975–1.101) | NA | [73] |
|  |  |  | East Asian | 8 | 3348 | 4019 | 0.953(0.877–1.036) | NA |  |
|  |  | RR vs CC | European | 7 | 2509 | 3082 | 1.012(0.831–1.233) | NA |  |
|  |  |  | East Asian | 8 | 2356 | 2732 | 1.031(0.803–1.324) | NA |  |
|  |  | Dominant | European | 7 | 4446 | 5315 | 1.071(0.988–1.161) | NA |  |
|  |  |  | East Asian | 8 | 3348 | 4019 | 0.929(0.842–1.024) | NA |  |
|  |  | **Recessive** | **European** | **7** | **4446** | **5315** | **0.985(0.864–1.123)** | **NA** |  |
|  |  |  | **East Asian** | **8** | **3348** | **4019** | **1.056(0.825–1.353)** | **NA** |  |
| MTHFR | rs1801133 | **'C vs R' OR 'Allele'** | **European** | **20** | **7404** | **8898** | **0.991(0.946–1.038)** | **0.96** | [73] |
|  |  |  | **East Asian** | **15** | **4370** | **6110** | **1.121(1.016–1.237)** | **NA** |  |
|  |  | RR vs CC | European | 20 | 4212 | 4998 | 0.99(0.854–1.148) | 1 |  |
|  |  |  | East Asian | 15 | 2410 | 3397 | 1.331(1.073–1.650) | NA |  |
|  |  | Dominant | European | 20 | 7404 | 8898 | 0.980(0.921–1.042) | NA |  |
|  |  |  | East Asian | 15 | 4370 | 6110 | 1.066(0.981–1.158) | NA |  |
|  |  | Recessive | European | 20 | 7404 | 8898 | 1.013(0.864–1.187) | 0.62 |  |
|  |  |  | East Asian | 15 | 4370 | 6110 | 1.265(1.058–1.513) | NA |  |
| MTR | A66G | RR vs CC | European | 2 | 1793 | 2306 | 1.01(0.87–1.16) | NA | [74] |
|  |  |  | Asian | 3 | 1571 | 1937 | 0.96(0.76–1.20) | NA |  |
|  |  | Dominant | European | 2 | 2391 | 3075 | 1.02 (0.90–1.16) | NA |  |
|  |  |  | Asian | 3 | 2095 | 2582 | 1.00(0.82–1.24) | NA |  |
|  |  | **Recessive** | **European** | **2** | **2391** | **3075** | **0.98(0.88–1.10)** | **NA** |  |
|  |  |  | **Asian** | **3** | **2095** | **2582** | **0.94(0.76–1.17)** | **NA** |  |
| NBS1 | rs1805794 | 'C vs R' OR 'Allele' | European | 7 | 3413 | 4600 | 0.95(0.86–1.04) | NA | [75] |
|  |  |  | African | 2 | 819 | 755 | 1.05(0.92–1.19) | NA |  |
|  |  | RR vs CC | European | 7 | 1948 | 2576 | 0.86 (0.67–1.12) | NA |  |
|  |  |  | African | 2 | 508 | 493 | 0.89(0.64–1.24) | NA |  |
|  |  | CC vs CR | European | 7 | 3079 | 4095 | 0.93(0.80–1.08) | NA |  |
|  |  |  | African | 2 | 776 | 708 | 1.14(0.92–1.41) | NA |  |
|  |  | Dominant | European | 7 | 3413 | 4600 | 0.92(0.77–1.08) | NA |  |
|  |  |  | African | 2 | 819 | 755 | 1.10(0.90–1.25) | NA |  |
|  |  | **Recessive** | **European** | **7** | **3413** | **4600** | **0.89(0.77–1.04)** | **NA** |  |
|  |  |  | **African** | **2** | **819** | **755** | **0.85(0.55–1.30)** | **NA** |  |
| NOS3 | rs1799983 | **RR vs CC** | **European** | **4** | **650** | **591** | **0.75(0.56-0.99)** | **NA** | [76] |
|  |  |  | **Asian** | **1** | **1145** | **793** | **0.13(0.02-1.01)** | **0.57** |  |
|  |  | CR vs RR | European | 4 | 701 | 593 | 0.82(0.62-1.08) | 1 |  |
|  |  |  | Asian | 1 | 214 | 152 | 0.12(0.02-0.96) | NA |  |
|  |  | Dominant | European | 4 | 1201 | 1079 | 0.79(0.60-1.02) | NA |  |
|  |  |  | Asian | 1 | 1348 | 944 | 0.13(0.02-1.00) | NA |  |
|  |  | Recessive | European | 4 | 1201 | 1079 | 0.88(0.75-1.04) | NA |  |
|  |  |  | Asian | 1 | 1348 | 944 | 1.02(0.81-1.28) | NA |  |
| NOS3 | rs2070744 | RR vs CC | European | 2 | 257 | 287 | 0.53(0.35-0.81) | NA | [76] |
|  |  |  | Asian | 1 | 1114 | 779 | 0.84(0.42-1.68) | 1 |  |
|  |  | CR vs RR | European | 2 | 303 | 274 | 0.70(0.46-1.07) | 1 |  |
|  |  |  | Asian | 1 | 272 | 190 | 0.84(0.41-1.70) | NA |  |
|  |  | Dominant | European | 2 | 492 | 514 | 0.61(0.41-0.91) | NA |  |
|  |  |  | Asian | 1 | 1364 | 956 | 0.84(0.42-1.68) | NA |  |
|  |  | **Recessive** | **European** | **2** | **492** | **514** | **0.70(0.55-0.91)** | **NA** |  |
|  |  |  | **Asian** | **1** | **1364** | **956** | **1.00(0.81-1.23)** | **0.11** |  |
| p21 | rs1801270 | RR vs CC | European | 16 | 15809 | 20980 | 1.496(1.164-1.924) | NA | [77] |
|  |  |  | Asian | 2 | 609 | 617 | 0.896(0.424-1.893) | 0.85 |  |
|  |  | CC vs CR | European | 16 | 15809 | 20980 | 1.037(0.962-1.119) | NA |  |
|  |  |  | Asian | 2 | 609 | 617 | 0.964(0.595-1.562) | NA |  |
|  |  | Dominant | European | 16 | 21079 | 27973 | 1.049(0.971-1.133) | NA |  |
|  |  |  | Asian | 2 | 812 | 823 | 0.941(0.537-1.650) | NA |  |
|  |  | **Recessive** | **European** | **16** | **21079** | **27973** | **1.492(1.161-1.919)** | **NA** |  |
|  |  |  | **Asian** | **2** | **812** | **823** | **0.911(0.592-1.403)** | **0.19** |  |
| RAD51 | rs1801320 | RR vs CC | European | 8 | 7444 | 5731 | 1.39(0.98–1.99) | NA | [78] |
|  |  |  | Asian | 3 | 849 | 883 | 1.37(0.75–2.52) | NA |  |
|  |  | **Dominant** | **European** | **8** | **9925** | **7641** | **0.90(0.71–1.15)** | **NA** |  |
|  |  |  | **Asian** | **3** | **1132** | **1177** | **1.02(0.84–1.25)** | **NA** |  |
|  |  | Recessive | European | 8 | 9925 | 7641 | 1.42(0.99–2.03) | NA |  |
|  |  |  | Asian | 3 | 1132 | 1177 | 1.41(0.77–2.59) | NA |  |
| RAS | rs191104666 | RR vs CC | European | 3 | 349 | 653 | 0.73(0.52–1.02) | 0.96 | [79] |
|  |  |  | Asian | 2 | 243 | 455 | 1.28(0.68–2.40) | 0.87 |  |
|  |  |  | African | 1 | 132 | 358 | 1.68(1.07–2.61) | NA |  |
|  |  | CC vs CR | European | 3 | 549 | 982 | 0.97(0.78–1.20) | NA |  |
|  |  |  | Asian | 2 | 387 | 766 | 0.98(0.76–1.26) | NA |  |
|  |  |  | African | 1 | 206 | 556 | 1.31(0.95–1.80) | NA |  |
|  |  | **Dominant** | **European** | **3** | **617** | **1133** | **0.92(0.75–1.12)** | **0.89** |  |
|  |  |  | **Asian** | **2** | **451** | **865** | **1.02(0.80–1.30)** | **0.78** |  |
|  |  |  | **African** | **1** | **248** | **634** | **1.39(1.03–1.88)** | **NA** |  |
|  |  | Recessive | European | 3 | 617 | 1133 | 0.77(0.56–1.05) | NA |  |
|  |  |  | Asian | 2 | 451 | 865 | 1.33(0.60–2.96) | NA |  |
|  |  |  | African | 1 | 248 | 634 | 1.45(0.97–2.18) | NA |  |
| RAS | rs4340 | RR vs CC | European | 7 | 570 | 3982 | 1.40(0.80–2.48) | 1 | [79] |
|  |  |  | Asian | 2 | 234 | 441 | 1.30(0.88–1.93) | 0.94 |  |
|  |  |  | African | 1 | 139 | 321 | 0.56 0.37–0.85 | NA |  |
|  |  | CC vs CR | European | 7 | 633 | 5557 | 0.91 0.66–1.23 | NA |  |
|  |  |  | Asian | 2 | 352 | 739 | 0.94(0.76–1.16) | NA |  |
|  |  |  | African | 1 | 180 | 410 | 0.91(0.70–1.18) | NA |  |
|  |  | **Dominant** | **European** | **7** | **985** | **7786** | **1.11(0.79–1.55)** | **1** |  |
|  |  |  | **Asian** | **2** | **408** | **821** | **0.98(0.76–1.25)** | **0.99** |  |
|  |  |  | **African** | **1** | **257** | **631** | **0.59(0.41–0.85)** | **NA** |  |
|  |  | Recessive | European | 7 | 985 | 7786 | 1.40(0.76–2.61) | NA |  |
|  |  |  | Asian | 2 | 408 | 821 | 1.35(0.94–1.96) | NA |  |
|  |  |  | African | 1 | 257 | 631 | 0.79(0.58–1.09) | NA |  |
| SOD2 | rs4880 | **'C vs R' OR 'Allele'** | **European** | **7** | **2708** | **3269** | **1.039 (0.965–1.120)** | **NA** |  |
|  |  |  | **Asian** | **2** | **1594** | **1936** | **1.036 (0.905–1.185)** | **NA** |  |
|  |  |  | **American** | **8** | **8596** | **9708** | **1.025 (0.948–1.109)** | **NA** |  |
|  |  | RR vs CC | European | 7 | 2031 | 2452 | 1.084 (0.929–1.265) | NA |  |
|  |  |  | Asian | 2 | 1196 | 1452 | 1.376 (0.863–2.196) | NA |  |
|  |  |  | American | 8 | 6447 | 7281 | 1.066 (0.898–1.265) | NA |  |
|  |  | Dominant | European | 7 | 2708 | 3269 | 1.166 (0.976–1.393) | NA |  |
|  |  |  | Asian | 2 | 1594 | 1936 | 1.010 (0.868–1.175) | NA |  |
|  |  |  | American | 8 | 8596 | 9708 | 0.984 (0.903–1.072) | NA |  |
|  |  | Recessive | European | 7 | 2708 | 3269 | 0.976 (0.859–1.109) | NA |  |
|  |  |  | Asian | 2 | 1594 | 1936 | 1.382 (0.868–2.202) | NA |  |
|  |  |  | American | 8 | 8596 | 9708 | 1.086 (0.936–1.259) | NA |  |
| SULT1A1 | rs9282861 | 'C vs R' OR 'Allele' | European | 9 | 5549 | 8382 | 1.00(0.96–1.05) | NA | [81] |
|  |  |  | Asian | 5 | 2905 | 3498 | 1.14(0.96–1.35) | NA |  |
|  |  | RR vs CC | European | 9 | 3119 | 4672 | 1.05(0.94–1.17) | 1 |  |
|  |  |  | Asian | 5 | 2410 | 2964 | 2.27(1.11 –4.63) | NA |  |
|  |  | CC vs CR | European | 9 | 4792 | 7276 | 1.08(0.93–1.25) | NA |  |
|  |  |  | Asian | 5 | 2887 | 3484 | 1.14(0.92–1.41) | NA |  |
|  |  | Dominant | European | 9 | 5549 | 8382 | 1.07(0.93–1.19) | NA |  |
|  |  |  | Asian | 5 | 2905 | 3498 | 1.17(0.94–1.46) | NA |  |
|  |  | **Recessive** | **European** | **9** | **5549** | **8382** | **0.98(0.82–1.16)** | **1** |  |
|  |  |  | **Asian** | **5** | **2905** | **3498** | **2.03(1.00 –4.41)** | **NA** |  |
| TGF-β1 | rs1800470 | RR vs CC | Asian | 5 | 1178 | 1607 | 1.02(0.87–1.20) | NA | [82] |
|  |  |  | African | 1 | 121 | 323 | 1.03(0.67–1.59) | NA |  |
|  |  | CC vs CR | Asian | 5 | 1766 | 2335 | 1.07(0.93–1.22) | NA |  |
|  |  |  | African | 1 | 188 | 494 | 1.05(0.74–1.47) | NA |  |
|  |  | **Dominant** | **Asian** | **5** | **2387** | **3183** | **1.05(0.92–1.20)** | **NA** |  |
|  |  |  | **African** | **1** | **233** | **612** | **1.04(0.75–1.43)** | **NA** |  |
|  |  | Recessive | Asian | 5 | 2387 | 3183 | 0.98(0.87–1.11) | NA |  |
|  |  |  | African | 1 | 233 | 612 | 1.00(0.68–1.47) | NA |  |
| TGF-β1 | rs1800470 | 'C vs R' OR 'Allele' | European | 7 | 4761 | 3946 | 1.056 (0.992–1.124) | NA | [83] |
|  |  |  | Asian | 5 | 2180 | 2464 | 1.028(0.947–1.117) | NA |  |
|  |  |  | American | 4 | 3451 | 5287 | 0.943(0.886–1.004) | NA |  |
|  |  | RR vs CC | European | 7 | 2594 | 2115 | 1.143(1.000–1.307) | NA |  |
|  |  |  | Asian | 5 | 1090 | 1250 | 1.035(0.875–1.225) | 0.29 |  |
|  |  |  | American | 4 | 1781 | 2747 | 0.865(0.734–1.020) | 0.53 |  |
|  |  | Dominant | European | 7 | 4761 | 3946 | 1.033(0.946–1.128) | NA |  |
|  |  |  | Asian | 5 | 2180 | 2464 | 1.083(0.860–1.362) | NA |  |
|  |  |  | American | 4 | 3451 | 5287 | 0.945(0.862–1.036) | NA |  |
|  |  | **Recessive** | **European** | **7** | **4761** | **3946** | **1.152(1.020 –1.301)** | **NA** |  |
|  |  |  | **Asian** | **5** | **2180** | **2464** | **1.022(0.891–1.172)** | **0.11** |  |
|  |  |  | **American** | **4** | **3451** | **5287** | **0.897(0.802–1.005)** | **0.16** |  |
| TNF-α | rs1800629 | 'C vs R' OR 'Allele' | European | 8 | 9595 | 12288 | 0.927(0.879-0.978) | NA | [84] |
|  |  |  | Asian | 3 | 358 | 575 | 1.731(0.450-6.654) | 0.07 |  |
|  |  |  | African | 2 | 283 | 280 | 1.642(0.827-3.261) | 0.07 |  |
|  |  | RR vs CC | European | 8 | 7113 | 8875 | 0.904(0.760-1.077) | 1 |  |
|  |  |  | Asian | 3 | 291 | 476 | 4.027(0.035-466.292) | 1 |  |
|  |  |  | African | 2 | 206 | 194 | 4.085(1.460-11.425) | NA |  |
|  |  | CR vs RR | European | 8 | 2720 | 3758 | 0.992(0.829-1.188) | 1 |  |
|  |  |  | Asian | 3 | 70 | 171 | 1.933 (0.338-11.060) | 1 |  |
|  |  |  | African | 2 | 101 | 91 | 4.861(1.746-13.527) | NA |  |
|  |  | **Dominant** | **European** | **8** | **9595** | **12288** | **0.915(0.861-0.972)** | **NA** |  |
|  |  |  | **Asian** | **3** | **358** | **575** | **1.774(0.447-7.037)** | **0.08** |  |
|  |  |  | **African** | **2** | **283** | **280** | **1.746(0.476-6.408)** | **0.07** |  |
|  |  | Recessive | European | 8 | 9595 | 12288 | 0.928(0.781-1.104) | 1 |  |
|  |  |  | Asian | 3 | 358 | 575 | 3.305(0.796-13.729) | 0.44 |  |
|  |  |  | African | 2 | 283 | 280 | 4.246(1.551-11.625) | NA |  |
| TP53 | rs1042522 | Dominant | European | 27 | 21017 | 22726 | 1.00 (0.90–1.10) | NA | [85] |
|  |  |  | Asian | 17 | 3611 | 5024 | 0.98 (0.84–1.14) | NA |  |
|  |  |  | Indians | 4 | 674 | 979 | 0.68 (0.45–1.03) | NA |  |
|  |  | **Recessive** | **European** | **27** | **21017** | **22726** | **0.97 (0.84–1.12** | **1** |  |
|  |  |  | **Asian** | **17** | **3611** | **5024** | **0.95 (0.79–1.15)** | **0.73** |  |
|  |  |  | **Indians** | **4** | **674** | **979** | **0.70 (0.50–0.92)** | **NA** |  |
|  |  | CC>CR>RR (additive) | European | 27 | 21017 | 22726 | 0.97 (0.84–1.12) | NA |  |
|  |  |  | Asian | 17 | 3611 | 5024 | 0.94 (0.74–1.20) | NA |  |
|  |  |  | Indians | 4 | 674 | 979 | 0.62 (0.46–1.82) | NA |  |
| TP53 | rs1625895 | Dominant | European | 10 | 8071 | 9306 | 1.01 (0.94–1.08) | NA | [85] |
|  |  |  | Mixed | 14 | 8787 | 9869 | 1.03 (0.91–1.18) | NA |  |
|  |  | Recessive | European | 10 | 8071 | 9306 | 0.93 (0.75–1.15) | NA |  |
|  |  |  | Mixed | 14 | 8787 | 9869 | 0.93 (0.76–1.14) | NA |  |
|  |  | **CC>CR>RR (additive)** | **European** | **10** | **8071** | **9306** | **0.93 (0.75–1.15)** | **NA** |  |
|  |  |  | **Mixed** | **14** | **8787** | **9869** | **0.93 (0.76–1.14)** | **NA** |  |
| TYMS | TS3'-UTR | 'C vs R' OR 'Allele' | European | 3 | 1157 | 1155 | 0.84 (0.53–1.36) | NA | [86] |
|  |  |  | Asian | 2 | 812 | 1135 | 1.08 (0.94–1.24) | NA |  |
|  |  | **RR vs CC** | **European** | **3** | **868** | **866** | **0.72 (0.24–2.19)** | **nd** |  |
|  |  |  | **Asian** | **2** | **447** | **661** | **1.41 (1.01–1.98)** | **NA** |  |
|  |  | CR vs RR | European | 3 | 868 | 866 | 0.74 (0.38–1.24) | NA |  |
|  |  |  | Asian | 2 | 752 | 1018 | 1.50 (0.92–2.45) | NA |  |
|  |  | Dominant | European | 3 | 1157 | 1155 | 0.91 (0.47–1.78) | NA |  |
|  |  |  | Asian | 2 | 812 | 1135 | 0.85 (0.65–1.11) | NA |  |
|  |  | Recessive | European | 3 | 1157 | 1155 | 0.76 (0.51–1.11) | NA |  |
|  |  |  | Asian | 2 | 812 | 1135 | 1.32 (0.94–1.83) | NA |  |
| TYMS | TSER 2R | 'C vs R' OR 'Allele' | European | 4 | 1823 | 2017 | 1.13 (1.02–1.25) | NA | [86] |
|  |  |  | Asian | 2 | 886 | 1383 | 1.01 (0.86–1.18) | 0.06 |  |
|  |  | RR vs CC | European | 4 | 927 | 1092 | 1.31 (1.10–1.57) | NA |  |
|  |  |  | Asian | 2 | 647 | 1001 | 1.22 (0.78–1.90) | 0.13 |  |
|  |  | CR vs RR | European | 4 | 1413 | 1601 | 1.13 (0.96–1.33) | NA |  |
|  |  |  | Asian | 2 | 847 | 1338 | 0.94 (0.78–1.14) | NA |  |
|  |  | Dominant | European | 4 | 1823 | 2017 | 1.19 (0.95–1.49) | NA |  |
|  |  |  | Asian | 2 | 886 | 1383 | 1.23 (0.79–1.91) | NA |  |
|  |  | **Recessive** | **European** | **4** | **1823** | **2017** | **1.17 (1.00–1.36)** | **NA** |  |
|  |  |  | **Asian** | **2** | **886** | **1383** | **0.97 (0.81–1.17)** | **0.05** |  |
| UGT1A1 | rs8175347 | **RR vs CC** | **European** | **3** | **2045** | **3422** | **0.88 (0.77-0.99)** | **NA** | [87] |
|  |  |  | **Asian** | **2** | **1204** | **1429** | **0.80 (0.47-1.38)** | **0.27** |  |
|  |  |  | **African** | **2** | **274** | **162** | **1.14 (0.46-2.80)** | **0.09** |  |
|  |  | CR vs RR | European | 3 | 2146 | 3532 | 0.90 (0.79-1.02) | NA |  |
|  |  |  | Asian | 2 | 339 | 414 | 0.74 (0.42-1.30) | NA |  |
|  |  |  | African | 2 | 378 | 238 | 0.96 (0.68-1.36) | NA |  |
|  |  | Dominant | European | 3 | 3707 | 6220 | 0.95 (0.88-1.04) | NA |  |
|  |  |  | Asian | 2 | 1515 | 1823 | 1.05 (0.89-1.24) | NA |  |
|  |  |  | African | 2 | 524 | 322 | 1.18 (0.52-2.65) | NA |  |
|  |  | Recessive | European | 3 | 3707 | 6220 | 0.89 (0.78-1.01) | NA |  |
|  |  |  | Asian | 2 | 1515 | 1823 | 0.79 (0.46-1.36) | NA |  |
|  |  |  | African | 2 | 524 | 322 | 1.03 (0.74-1.42) | NA |  |
| VEGF | rs2010963 | RR vs CC | European | 12 | 3581 | 3642 | 0.93 (0.83–1.05) | NA | [88] |
|  |  |  | Asian | 10 | 2194 | 2283 | 1.06 (0.81–1.38) | NA |  |
|  |  |  | African | 1 | 44 | 54 | 0.44 (0.17–1.11) | NA |  |
|  |  | CR vs RR | European | 12 | 3526 | 3466 | 0.98 (0.87–1.10) | NA |  |
|  |  |  | Asian | 10 | 3001 | 3088 | 1.08 (0.84–1.39) | NA |  |
|  |  |  | African | 1 | 72 | 56 | 0.83 (0.34–2.01) | NA |  |
|  |  | **Dominant** | **European** | **12** | **6332** | **6455** | **1.11 (0.90–1.36)** | **NA** |  |
|  |  |  | **Asian** | **10** | **4448** | **4581** | **1.01 (0.85–1.21)** | **NA** |  |
|  |  |  | **African** | **1** | **101** | **100** | **1.63 (0.92–2.90)** | **NA** |  |
|  |  | Recessive | European | 12 | 6332 | 6455 | 0.96 (0.86–1.07) | NA |  |
|  |  |  | Asian | 10 | 4448 | 4581 | 1.07 (0.84–1.35) | NA |  |
|  |  |  | African | 1 | 101 | 100 | 0.64 (0.27–1.50) | NA |  |
| VEGF | rs3025039 | RR vs CC | European | 8 | 3300 | 3254 | 1.07 (0.79–1.44) | NA | [89] |
|  |  |  | Asian | 2 | 997 | 1147 | 0.72 (0.47–1.09) | NA |  |
|  |  | CC vs CR | European | 8 | 3300 | 3254 | 0.93 (0.75–1.16) | NA |  |
|  |  |  | Asian | 2 | 997 | 1147 | 0.94 (0.80–1.11) | NA |  |
|  |  | **Dominant** | **European** | **8** | **4400** | **4339** | **0.94 (0.76–1.16)** | **NA** |  |
|  |  |  | **Asian** | **2** | **1329** | **1529** | **0.92 (0.78–1.07)** | **NA** |  |
|  |  | Recessive | European | 8 | 4400 | 4339 | 1.10 (0.82–1.48) | NA |  |
|  |  |  | Asian | 2 | 1329 | 1529 | 0.72 (0.48–1.10) | NA |  |
| XPF | rs1800067 | RR vs CC | European | 4 | 1792 | 1810 | 5.20 (2.08–12.95) | NA | [90] |
|  |  |  | African | 2 | 790 | 715 | 2.61 (0.11–64.18) | 1 |  |
|  |  | CC vs CR | European | 4 | 2055 | 2077 | 1.08 (0.90–1.29) | 1 |  |
|  |  |  | African | 2 | 809 | 748 | 0.55 (0.31–0.96) | NA |  |
|  |  | Dominant | European | 4 | 2082 | 2082 | 1.16 (0.98–1.39) | NA |  |
|  |  |  | African | 2 | 810 | 748 | 0.58 (0.33–1.00) | NA |  |
|  |  | **Recessive** | **European** | **4** | **2082** | **2082** | **5.15 (2.07–12.83)** | **NA** |  |
|  |  |  | **African** | **2** | **810** | **748** | **2.67 (0.11–65.67)** | **0.13** |  |
| XPG | rs17655 | RR vs CC | European | 4 | 1749 | 1912 | 0.99 (0.78–1.26) | NA | [90] |
|  |  |  | Asian | 1 | 210 | 288 | 0.70 (0.49–1.01) | NA |  |
|  |  |  | African | 2 | 390 | 392 | 1.06 (0.80–1.42) | NA |  |
|  |  | CC vs CR | European | 4 | 2510 | 2739 | 1.00 (0.89–1.12) | NA |  |
|  |  |  | Asian | 1 | 325 | 402 | 0.93 (0.69–1.26) | NA |  |
|  |  |  | African | 2 | 663 | 606 | 1.21 (0.96–1.52) | NA |  |
|  |  | Dominant | European | 4 | 2652 | 2895 | 1.00 (0.90–1.11) | NA |  |
|  |  |  | Asian | 1 | 401 | 531 | 0.85 (0.65–1.13) | NA |  |
|  |  |  | African | 2 | 809 | 749 | 1.17 (0.94–1.45) | NA |  |
|  |  | **Recessive** | **European** | **4** | **2652** | **2895** | **0.99 (0.79–1.26)** | **NA** |  |
|  |  |  | **Asian** | **1** | **401** | **531** | **0.73 (0.53–1.00)** | **NA** |  |
|  |  |  | **African** | **2** | **809** | **749** | **0.86 (0.67–1.11)** | **NA** |  |
