# Supplementary material for: Ethnic Background and Genetic Variation in the Evaluation of Cancer Risk: A Systematic Review
Source: PLoS One. 2014 Jun 5;9(6):e97522. doi: 10.1371/journal.pone.0097522 (PMC4046957; doi:10.1371/journal.pone.0097522)
Supplement: Tables S3 — The association of the assessed variations with risk of colon cancer [92]–[102]. (DOCX) [file pone.0097522.s006.docx]

| Table S3 Associations with colon cancer**Gene** | **SNP** | **Model** | **Ethnicity** | **# of studies** | **# of cases** | **# of controls** | **Odd's Ratio** | **Power** | **Reference** |
| --- | --- | --- | --- | --- | --- | --- | --- | --- | --- |
| CCND1 | rs603965 | RR vs CC | European | 17 | 1836 | 2236 | 1.27 (1.04– 1.44) | NA | [91] |
|  |  |  | Asian | 7 | 1131 | 1994 | 1.15 (0.86– 1.54) | 0.66 |  |
|  |  | CR vs RR | European | 17 | 2854 | 3368 | 1.12 (1.00– 1.27) | NA |  |
|  |  |  | Asian | 7 | 1834 | 3095 | 1.13 (0.86– 1.49) | 0.31 |  |
|  |  | **Dominant** | **European** | **17** | **3747** | **4487** | **1.17 (1.02 – 1.34)** | **NA** |  |
|  |  |  | **Asian** | **7** | **2228** | **3828** | **1.13 (0.87 – 1.48)** | **0.6** |  |
| Chr. 8 | rs10505477 | 'C vs R' OR 'Allele' | European | 9 | 17566 | 15976 | 1.02(0.84-1.23) | NA | [92] |
|  |  |  | Americans | 7 | 7999 | 9276 | 0.90(0.86-0.74) | NA |  |
|  |  | RR vs CC | European | 9 | 8879 | 8004 | 1.03(0.70-1.52) | 1 |  |
|  |  |  | Americans | 7 | 4171 | 4930 | 0.80(0.73-0.88) | NA |  |
|  |  | CR vs RR | European | 9 | 13467 | 11926 | 1.02(0.83-1.24) | 1 |  |
|  |  |  | Americans | 7 | 5404 | 6376 | 0.86(0.79-0.93) | NA |  |
|  |  | Dominant | European | 9 | 17566 | 15976 | 1.03(0.79-1.33) | 1 |  |
|  |  |  | Americans | 7 | 7999 | 9276 | 0.89(0.80-0.99) | NA |  |
|  |  | Recessive | European | 9 | 17566 | 15976 | 0.98(0.76-1.27) | 0.59 |  |
|  |  |  | Americans | 7 | 7999 | 9276 | 1.19 (1.10-1.29) | NA |  |
| Chr. 8 | rs6983267 | RR vs CC | European | 9 | 10456 | 9952 | 0.74(0.62-0.89) | NA | [92] |
|  |  |  | Asian | 4 | 4809 | 4467 | 0.69(0.63-0.75) | NA |  |
|  |  |  | Americans | 7 | 6302 | 5797 | 0.90(0.73-1.11) | 1 |  |
|  |  | CR vs RR | European | 9 | 14658 | 14637 | 0.85(0.75-0.97) | NA |  |
|  |  |  | Asian | 4 | 7658 | 7236 | 0.85(0.80-0.91) | NA |  |
|  |  |  | Americans | 7 | 5528 | 7462 | 0.93(0.82-1.05) | 0.99 |  |
|  |  | **Dominant** | **European** | **9** | **21090** | **19871** | **0.83(0.74-0.92)** | **NA** |  |
|  |  |  | **Asian** | **4** | **9191** | **8365** | **0.75(0.69-0.81)** | **NA** |  |
|  |  |  | **Americans** | **7** | **8238** | **10889** | **0.96(0.83-1.10)** | **1** |  |
|  |  | Recessive | European | 9 | 21090 | 19871 | 0.81(0.70-0.94) | NA |  |
|  |  |  | Asian | 4 | 9191 | 8365 | 0.81(0.76-0.86) | NA |  |
|  |  |  | Americans | 7 | 8238 | 10889 | 0.92(0.79-1.07) | 0.64 |  |
| COX-2 | rs20417 | RR vs CC | European | 8 | 1558 | 1637 | 1.02 (0.73-1.43) | NA | [93] |
|  |  |  | Asian | 12 | 2974 | 4732 | 2.03 (0.42-9.87) | NA |  |
|  |  | CC vs CR | European | 8 | 1558 | 1637 | 0.92 (0.80-1.06) | NA |  |
|  |  |  | Asian | 12 | 2974 | 4732 | 1.37 (0.86-2.19) | NA |  |
|  |  | Dominant | European | 8 | 2077 | 2183 | 0.93 (0.81-1.06) | NA |  |
|  |  |  | Asian | 12 | 3965 | 6309 | 1.41 (0.91-2.18) | NA |  |
|  |  | **Recessive** | **European** | **8** | **2077** | **2183** | **1.04 (0.74-1.44)** | **NA** |  |
|  |  |  | **Asian** | **12** | **3965** | **6309** | **1.87 (0.51- 6.78)** | **NA** |  |
| COX-2 | rs5275 | RR vs CC | European | 5 | 1400 | 1872 | 1.20 (0.83-1.75) | NA |  |
|  |  |  | Asian | 3 | 561 | 884 | 1.20 (0.83-1.74) | NA |  |
|  |  | CC vs CR | European | 5 | 1400 | 1872 | 1.00 (0.88-1.14) | NA |  |
|  |  |  | Asian | 3 | 561 | 884 | 1.19 (0.97-1.46) | NA |  |
|  |  | **Dominant** | **European** | **5** | **1866** | **1496** | **1.03 (0.91-1.16)** | **NA** |  |
|  |  |  | **Asian** | **3** | **748** | **1178** | **1.20 (0.99-1.46)** | **NA** |  |
|  |  | Recessive | European | 5 | 1866 | 1496 | 1.12 (0.93-1.35) | NA |  |
|  |  |  | Asian | 3 | 748 | 1178 | 1.07 (0.76- 1.50) | NA |  |
| COX-2 | rs689466 | RR vs CC | European | 4 | 1217 | 1627 | 0.91 (0.47- 1.76) | 1 | [93] |
|  |  |  | Asian | 8 | 2667 | 3614 | 1.75 (1.53-1.99) | NA |  |
|  |  | CC vs CR | European | 4 | 1217 | 1627 | 0.93 (0.66-1.30) | 0.85 |  |
|  |  |  | Asian | 8 | 2667 | 3614 | 1.27 (1.13-1.42) | NA |  |
|  |  | Dominant | European | 4 | 1622 | 2169 | 0.90 (0.48-1.67) | 1 |  |
|  |  |  | Asian | 8 | 3556 | 4819 | 1.41 (1.27-1.57) | NA |  |
|  |  | **Recessive** | **European** | **4** | **1622** | **2169** | **1.08 (0.95-1.24)** | **0.31** |  |
|  |  |  | **Asian** | **8** | **3556** | **4819** | **1.50 (1.35-1.66)** | **NA** |  |
| CYP1A1 | rs1048943 | RR vs CC | European | 6 | 2224 | 2416 | 1.67 (0.87–3.19) | 0.81 | [94] |
|  |  |  | Asian | 5 | 1260 | 1673 | 1.38 (1.07–1.79) | NA |  |
|  |  | CC vs CR | European | 6 | 2224 | 2416 | 1.24 (1.05–1.46) | NA |  |
|  |  |  | Asian | 5 | 1260 | 1673 | 0.90 (0.78–1.04) | 0.81 |  |
|  |  | Dominant | European | 6 | 2992 | 3221 | 1.26 (1.07–1.48) | NA |  |
|  |  |  | Asian | 5 | 1680 | 2231 | 0.97 (0.85–1.11) | 0.94 |  |
|  |  | **Recessive** | **European** | **6** | **2992** | **3221** | **1.62 (0.84–3.10)** | **0.07** |  |
|  |  |  | **Asian** | **5** | **1680** | **2231** | **1.44 (1.12–1.86)** | **NA** |  |
| CYP1A1 | rs4646903 | RR vs CC | European | 7 | 1942 | 2157 | 1.18 (0.52–2.66) | NA | [94] |
|  |  |  | Asian | 3 | 668 | 932 | 0.88 (0.67–1.16) | NA |  |
|  |  | CC vs CR | European | 7 | 1942 | 2157 | 1.01 (0.88–1.16) | NA |  |
|  |  |  | Asian | 3 | 668 | 932 | 0.92 (0.64–1.32) | NA |  |
|  |  | Dominant | European | 7 | 2589 | 2876 | 1.01 (0.88–1.16) | NA |  |
|  |  |  | Asian | 3 | 891 | 1242 | 0.91 (0.62–1.33) | NA |  |
|  |  | **Recessive** | **European** | **7** | **2589** | **2876** | **1.19 (0.52–2.69)** | **NA** |  |
|  |  |  | **Asian** | **3** | **891** | **1242** | **0.94 (0.73–1.21)** | **NA** |  |
| FTO | rs1421085 | RR vs CC | European | 1 | 87 | 286 | 0.84(0.49-1.44) | NA | [95] |
|  |  |  | African American | 1 | 108 | 263 | no | NA |  |
|  |  | CC vs CR | European | 1 | 157 | 466 | 1.06(0.72-1.56) | NA |  |
|  |  |  | African American | 1 | 134 | 326 | 1.05 (0.62-1.78) | NA |  |
|  |  | **Dominant** | **European** | **1** | **184** | **572** | **1.00(0.69-1.45)** | **NA** |  |
|  |  |  | **African American** | **1** | **137** | **329** | **1.15(0.69-1.92)** | **NA** |  |
| FTO | rs17817449 | RR vs CC | European | 1 | 83 | 286 | 0.84(0.48-1.47) | NA | [95] |
|  |  |  | African American | 1 | 83 | 158 | 0.85(0.46-1.57) | NA |  |
|  |  | CC vs CR | European | 1 | 160 | 473 | 1.18(0.80-1.74) | 0.92 |  |
|  |  |  | African American | 1 | 112 | 277 | 0.54(0.34-0.87) | NA |  |
|  |  | **Dominant** | **European** | **1** | **184** | **571** | **1.10 (0.76-1.59)** | **0.82** |  |
|  |  |  | **African American** | **1** | **137** | **329** | **0.61 (0.39-0.95)** | **NA** |  |
| FTO | rs8044769 | RR vs CC | European | 1 | 80 | 281 | 1.04(0.62-1.76) | NA | [95] |
|  |  |  | African American | 1 | 93 | 208 | 0.91(0.39-2.12) | NA |  |
|  |  | CC vs CR | European | 1 | 145 | 447 | 1.26(0.83-1.94) | NA |  |
|  |  |  | African American | 1 | 126 | 305 | 0.85(0.54-1.35) | NA |  |
|  |  | **Dominant** | **European** | **1** | **183** | **569** | **1.20(0.80-1.80)** | **NA** |  |
|  |  |  | **African American** | **1** | **137** | **328** | **0.86(0.56-1.33)** | **NA** |  |
| FTO | rs8050136 | RR vs CC | European | 1 | 83 | 286 | 0.85(0.49-1.49) | NA | [95] |
|  |  |  | African American | 1 | 83 | 158 | 0.75(0.41-1.36) | NA |  |
|  |  | CC vs CR | European | 1 | 160 | 473 | 1.19 (0.81-1.75) | 0.92 |  |
|  |  |  | African American | 1 | 109 | 264 | 0.54(0.33-0.87) | NA |  |
|  |  | **Dominant** | **European** | **1** | **184** | **572** | **1.10 (0.76-1.60)** | **0.87** |  |
|  |  |  | **African American** | **1** | **137** | **329** | **0.59(0.38-0.93)** | **NA** |  |
| FTO | rs9939609 | RR vs CC | European | 1 | 85 | 287 | 0.87(0.50-1.52) | NA | [95] |
|  |  |  | African American | 1 | 76 | 153 | 0.81(0.45-1.46) | NA |  |
|  |  | **CC vs CR** | **European** | **1** | **159** | **476** | **1.14(0.77-1.67)** | **0.86** |  |
|  |  |  | **African American** | **1** | **103** | **253** | **0.57(0.35-0.96)** | **NA** |  |
|  |  | Dominant | European | 1 | 184 | 573 | 1.07(0.74-1.55) | NA |  |
|  |  |  | African American | 1 | 137 | 330 | 0.64(0.40-1.03) | NA |  |
| MMP1 | 1607 1G/2G | Dominant | European | 5 | 991 | 1197 | 1.52 (1.25– 1.85) | NA | [96] |
|  |  |  | Asian | 3 | 412 | 557 | 1.70 (1.01– 2.85) | NA |  |
|  |  | **Recessive** | **European** | **5** | **991** | **1197** | **1.52 (1.25– 1.85)** | **NA** |  |
|  |  |  | **Asian** | **3** | **412** | **557** | **1.48 (0.88– 2.48)** | **0.08** |  |
| MMP1 | rs3025058 | Dominant | European | 2 | 250 | 536 | 1.13 (0.79– 1.61) | NA | [96] |
|  |  |  | Asian | 3 | 405 | 498 | 0.52 (0.20– 1.34) | NA |  |
|  |  | **Recessive** | **European** | **2** | **250** | **536** | **1.25 (0.84– 1.86)** | **NA** |  |
|  |  |  | **Asian** | **3** | **405** | **498** | **1.12 (0.57– 2.20)** | **NA** |  |
| MTHFR | rs1801131 | Dominant | European | 1 | 309 | 541 | 0.8 (0.3–2.0) | NA | [97] |
|  |  |  | African American | 1 | 243 | 329 | 1.1 (0.7–1.5) | NA |  |
|  |  | **Recessive** | **European** | **1** | **309** | **541** | **0.5 (0.3–0.9)** | **NA** |  |
|  |  |  | **African American** | **1** | **243** | **329** | **0.8 (0.6–1.0)** | **0.17** |  |
| MTHFR | rs1801133 | **RR vs CC** | **European** | **14** | **4167** | **6383** | **0.83(0.74-0.94)** | **NA** | [98] |
|  |  |  | **Asian** | **8** | **1477** | **2444** | **0.80(0.67-0.96)** | **NA** |  |
|  |  | CC vs CR | European | 14 | 4167 | 6383 | 0.97(0.86-1.09) | NA |  |
|  |  |  | Asian | 8 | 1477 | 2444 | 0.97(0.86-1.10) | NA |  |
| MTR | rs1805087 | 'C vs R' OR 'Allele' | European | 3 | 724 | 932 | 0.86 (0.77–0.95) | NA | [99] |
|  |  |  | Asian | 3 | 714 | 1406 | 1.03 (0.87–1.21) | 0.31 |  |
|  |  |  | others | 6 | 3960 | 5367 | 1.04 (0.96–1.13) | NA |  |
|  |  | RR vs CC | European | 3 | 498 | 653 | 0.61 (0.45–0.84) | NA |  |
|  |  |  | Asian | 3 | 498 | 961 | 1.30 (0.81–2.08) | 0.98 |  |
|  |  |  | others | 6 | 2661 | 3694 | 0.94 (0.74–1.19) | NA |  |
|  |  | CC vs CR | European | 3 | 694 | 893 | 0.90 (0.80–1.02) | NA |  |
|  |  |  | Asian | 3 | 683 | 1359 | 0.96 (0.79–1.18) | NA |  |
|  |  |  | others | 6 | 3810 | 5157 | 1.09 (0.99–1.20) | NA |  |
|  |  | Dominant | European | 3 | 724 | 932 | 0.87 (0.77–0.98) | NA |  |
|  |  |  | Asian | 3 | 714 | 1406 | 1.00 (0.82–1.21) | 0.29 |  |
|  |  |  | others | 6 | 3960 | 5367 | 1.07 (0.98–1.18) | NA |  |
|  |  | **Recessive** | **European** | **3** | **724** | **932** | **0.63 (0.47–0.87)** | **NA** |  |
|  |  |  | **Asian** | **3** | **714** | **1406** | **1.32 (0.83–2.10)** | **0.23** |  |
|  |  |  | others | **6** | **3960** | **5367** | **0.91 (0.72–1.16)** | **NA** |  |
| TGFB1 | rs1800469 | RR vs CC | European | 1 | 94 | 234 | 0.80(0.42-1.54) | 0.62 | [100] |
|  |  |  | Asian | 4 | 451 | 964 | 1.77(1.40-2.24) | NA |  |
|  |  | CR vs RR | European | 1 | 79 | 238 | 0.63(0.33-1.23) | NA |  |
|  |  |  | Asian | 4 | 559 | 1459 | 1.18(0.95-1.46) | NA |  |
|  |  | **Dominant** | **European** | **1** | **157** | **439** | **1.17(0.81-1.68)** | **0.67** |  |
|  |  |  | **Asian** | **4** | **837** | **1896** | **1.58(1.31-1.89)** | **NA** |  |
|  |  | Recessive | European | 1 | 157 | 439 | 0.72(0.38-1.34) | 0.08 |  |
|  |  |  | Asian | 4 | 837 | 1896 | 1.38(1.13-1.68) | NA |  |
| TP53 | rs1042522 | RR vs CC | European | 9 | 1451 | 2094 | 0.80 (0.60-1.07) | NA | [101] |
|  |  |  | Asian | 7 | 780 | 1505 | 1.23 (0.80-1.90) | NA |  |
|  |  | CC vs CR | European | 9 | 1451 | 2094 | 1.02 (0.81–1.28) | NA |  |
|  |  |  | Asian | 7 | 780 | 1505 | 1.13 (0.96-1.34) | NA |  |
|  |  | Dominant | European | 9 | 1934 | 2792 | 0.98 (0.80-1.21) | NA |  |
|  |  |  | Asian | 7 | 1040 | 2006 | 1.12 (0.87-1.43) | NA |  |
|  |  | **Recessive** | **European** | **9** | **1934** | **2792** | **0.81 (0.61-1.07)** | **NA** |  |
|  |  |  | **Asian** | **7** | **1040** | **2006** | **1.19 (0.83-1.71)** | **NA** |  |
| XRCC1 | rs1799782 | **'C vs R' OR 'Allele'** | **European** | **4** | **728** | **943** | **0.96(0.82-1.13)** | **NA** | [102] |
|  |  |  | **Asian** | **2** | **514** | **1371** | **0.99 (0.84–1.16)** | **NA** |  |
|  |  | RR vs CC | European | 4 | 649 | 828 | 2.75(0.91-8.29) | NA |  |
|  |  |  | Asian | 2 | 303 | 822 | 0.96(0.67-1.39) | NA |  |
|  |  | Dominant | European | 4 | 728 | 943 | 0.96(0.71-1.29) | NA |  |
|  |  |  | Asian | 2 | 514 | 1371 | 1.00(0.81-1.23) | NA |  |
|  |  | Recessive | European | 4 | 728 | 943 | 2.81(0.93-8.46) | NA |  |
|  |  |  | Asian | 2 | 514 | 1371 | 0.94(0.66-1.34) | NA |  |
| XRCC1 | rs25487 | 'C vs R' OR 'Allele' | European | 5 | 772 | 1043 | 0.95(0.82-1.09) | NA | [102] |
|  |  |  | Asian | 3 | 1221 | 2058 | 0.94(0.84-1.06) | NA |  |
|  |  | RR vs CC | European | 5 | 432 | 560 | 0.93(0.69-1.26) | NA |  |
|  |  |  | Asian | 3 | 761 | 1275 | 0.83(0.61-1.13) | NA |  |
|  |  | **Dominant** | **European** | **5** | **772** | **1043** | **0.91(0.75-1.10)** | **NA** |  |
|  |  |  | **Asian** | **3** | **1221** | **2058** | **0.95(0.82-1.10)** | **NA** |  |
|  |  | Recessive | European | 5 | 772 | 1043 | 0.99(0.75-1.32) | NA |  |
|  |  |  | Asian | 3 | 1221 | 2058 | 0.85(0.63-1.15) | NA |  |
| XRCC3 | Thr241Met | **RR vs CC** | **European** | **4** | **791** | **1052** | **1.04(1.85-1.28)** | **NA** | [102] |
|  |  |  | **Asian** | **2** | **786** | **929** | **0.90(0.15-5.24)** | **0.06** |  |
|  |  | Dominant | European | 4 | 1462 | 1997 | 0.95(0.83-1.09) | NA |  |
|  |  |  | Asian | 2 | 861 | 1014 | 0.99(0.72-1.37) | NA |  |
|  |  | Recessive | European | 4 | 1462 | 1997 | 1.00(0.83-1.22) | NA |  |
|  |  |  | Asian | 2 | 861 | 1014 | 0.88(0.15-5.14) | NA |  |
