# Supplementary material for: Ethnic Background and Genetic Variation in the Evaluation of Cancer Risk: A Systematic Review
Source: PLoS One. 2014 Jun 5;9(6):e97522. doi: 10.1371/journal.pone.0097522 (PMC4046957; doi:10.1371/journal.pone.0097522)
Supplement: Tables S4 — The association of the assessed variations with risk of liver cancer [103]–[106]. (DOCX) [file pone.0097522.s007.docx]

Table S4 Associations with liver cancer

| **Gene** | **SNP** | **Model** | **Ethnicity** | **# of studies** | **# of cases** | **# of controls** | **Odd's Ratio** | **Power** | **Reference** |
| --- | --- | --- | --- | --- | --- | --- | --- | --- | --- |
| GSTM1 | null genotype | **'C vs R' OR 'Allele'** | **European** | **4** | **478** | **930** | **0.93(0.74–1.16)** | **nd** | [103] |
|  |  |  | **Asian** | **18** | **2651** | **4309** | **1.36(1.06–1.76)** | **NA** |  |
|  |  |  | **African** | **2** | **220** | **370** | **1.18(0.82–1.70)** | **nd** |  |
| GSTT1 | null genotype | **'C vs R' OR 'Allele'** | **European** | **3** | **422** | **841** | **1.05(0.79–1.39)** | **nd** | [103] |
|  |  |  | **Asian** | **12** | **1994** | **3199** | **1.40(1.17–1.68)** | **NA** |  |
|  |  |  | **African** | **2** | **220** | **370** | **1.06(0.76–1.49)** | **nd** |  |
| HLA-DRB1 | HLA-DRB1*07 | **C vs R' OR 'Allele'** | **Asian** | **3** | **156** | **224** | **2.10(1.06-4.14)** | **NA** | [104] |
|  |  |  | **others** | **3** | **125** | **242** | **1.41(0.83-2.42)** | **NA** |  |
| HLA-DRB1 | DRB1*15 | **'C vs R' OR 'Allele'** | **Asian** | **3** | **156** | **224** | **3.22(1.63-6.37)** | **NA** | [104] |
|  |  |  | **others** | **3** | **125** | **242** | **0.80(0.34-1.89)** | **NA** |  |
| TNFA | rs361525 | RR vs CC | European | 1 | 23 | 72 | nd | NA | [105] |
|  |  |  | Asain | 7 | 681 | 956 | 1.44(0.33-6.24) | NA |  |
|  |  | CC vs CR | European | 1 | 23 | 72 | nd | NA |  |
|  |  |  | Asain | 7 | 681 | 956 | 1.63(1.17-2.22) | NA |  |
|  |  | **Dominant** | **European** | **1** | **30** | **96** | **1.69 (0.47-6.07)** | **0.15** |  |
|  |  |  | **Asain** | **7** | **908** | **1274** | **1.61 (1.16-2.24)** | **NA** |  |
| TNFA | rs1800629 | **Dominant** | **European** | **4** | **270** | **484** | **1.54(0.62-3.87)** | **0.98** | [105] |
|  |  |  | **Asian** | **10** | **1565** | **1973** | **1.88(1.14-3.10)** | **NA** |  |
|  |  | Recessive | European | 4 | 270 | 484 | 1.57(0.45-5.47) | NA |  |
|  |  |  | Asian | 10 | 1565 | 1973 | 1.91(0.87-4.22) | NA |  |
| TNFA | C863A | RR vs CC | European | 1 | 23 | 72 | nd | NA | [105] |
|  |  |  | Asian | 4 | 448 | 681 | 1.40 (0.74-2.63) | NA |  |
|  |  | CC vs CR | European | 1 | 23 | 72 | nd | NA |  |
|  |  |  | Asian | 4 | 448 | 681 | 1.72 (1.03-2.88) | NA |  |
|  |  | **Dominant** | **European** | **1** | **30** | **96** | **1.40 (0.60-3.26)** | **0.23** |  |
|  |  |  | **Asian** | **4** | **597** | **908** | **1.71 (1.02-2.86)** | **NA** |  |
| XRCC1 | rs25487 | RR vs CC | Asian | 9 | 1452 | 2076 | 1.05 (0.82-1.35) | NA | [106] |
|  |  |  | Non-Asian | 2 | 204 | 373 | 0.64 (0.28-1.45) | NA |  |
|  |  | CR vs RR | Asian | 9 | 1452 | 2076 | 1.15 (0.84-1.59) | NA |  |
|  |  |  | Non-Asian | 2 | 204 | 373 | 0.81 (0.32-2.03) | NA |  |
|  |  | **Dominant** | **Asian** | **9** | **1936** | **2768** | **1.19 (0.89-1.59)** | **NA** |  |
|  |  |  | **Non-Asian** | **2** | **272** | **497** | **0.80 (0.29-2.19)** | **NA** |  |
|  |  | Recessive | Asian | 9 | 1936 | 2768 | 1.01 (0.79-1.29) | NA |  |
|  |  |  | Non-Asian | 2 | 272 | 497 | 0.80 (0.37-1.74) | NA |  |
