# Supplementary material for: Ethnic Background and Genetic Variation in the Evaluation of Cancer Risk: A Systematic Review
Source: PLoS One. 2014 Jun 5;9(6):e97522. doi: 10.1371/journal.pone.0097522 (PMC4046957; doi:10.1371/journal.pone.0097522)
Supplement: Tables S5 — The association of the assessed variations with risk of lung cancer [107]–[110]. (DOCX) [file pone.0097522.s008.docx]

Table S5 Associations with gastric cancer

| **Gene** | **SNP** | **Model** | **Ethnicity** | **# of studies** | **# of cases** | **# of controls** | **Odd's Ratio** | **Power** | **Reference** |
| --- | --- | --- | --- | --- | --- | --- | --- | --- | --- |
| CDH1 | rs16260 | C vs R' OR 'Allele' | European | 8 | 1521 | 2434 | 0.79 (0.64-0.96) | NA | [107] |
|  |  |  | Asian | 9 | 1990 | 2392 | 1.10 (0.97-1.24) | 0.94 |  |
|  |  | RR vs CC | European | 8 | 868 | 1400 | 0.61 (0.40-0.93) | NA |  |
|  |  |  | Asian | 9 | 1319 | 1580 | 1.23 (0.79-1.90) | 1.00 |  |
|  |  | CR vs RR | European | 8 | 811 | 1230 | 1.27 (0.99-1.62) | NA |  |
|  |  |  | Asian | 9 | 769 | 934 | 0.95 (0.58-1.55) | NA |  |
|  |  | CC vs CR | European | 8 | 1359 | 2234 | 1.15 (0.92-1.44) | NA |  |
|  |  |  | Asian | 9 | 1892 | 2270 | 0.92 (0.81-1.05) | NA |  |
|  |  | Dominant | European | 8 | 1521 | 2434 | 0.80 (0.63-1.01) | NA |  |
|  |  |  | Asian | 9 | 1990 | 2392 | 1.13 (0.95-1.33) | NA |  |
|  |  | **Recessive** | **European** | **8** | **1521** | **2434** | **1.50 (1.03-2.19)** | **NA** |  |
|  |  |  | **Asian** | **9** | **1990** | **2392** | **0.87 (0.56-1.37)** | **0.45** |  |
| IL1beta | A511T | **Dominant** | **European** | **8** | **1463** | **1850** | **1.49 (1.20-1.85)** | **NA** | [108] |
|  |  |  | **Asian** | **7** | **1517** | **1644** | **0.95 (0.81-1.13)** | **1.00** |  |
| XRCC1 | rs1799782 | Allelic | European | 2 | 540 | 1721 | 1.41(0.89-2.23) | NA | [109] |
|  |  |  | Asian | 7 | 1719 | 2203 | 0.84(0.64-1.12) | na |  |
|  |  | RR vs CC | European | 2 | 484 | 1515 | 2.16(0.27-16.93) | NA |  |
|  |  |  | Asian | 7 | 975 | 1197 | 0.76(0.45-1.29) | NA |  |
|  |  | CR vs RR | European | 2 | 21 | 136 | 1.59(0.19-13.08) | NA |  |
|  |  |  | Asian | 7 | 613 | 1067 | 0.79(0.61-1.00) | NA |  |
|  |  | CC vs CR | European | 2 | 503 | 1631 | 1.36(0.83-2.22) | NA |  |
|  |  |  | Asian | 7 | 1250 | 1942 | 0.86(0.61-1.21) | NA |  |
|  |  | Dominant | European | 2 | 540 | 1721 | 1.31(0.95-1.79) | NA |  |
|  |  |  | Asian | 7 | 1719 | 2203 | 0.84(0.58-1.20) | NA |  |
|  |  | **Recessive** | **European** | **2** | **540** | **1721** | **0.48(0.06-3.75)** | **0.06** |  |
|  |  |  | **Asian** | **7** | **1719** | **2203** | **1.35(1.07-1.70)** | **NA** |  |
| XRCC3 | rs861539 | RR vs CC | European | 3 | 251 | 536 | 1.46(1.00-2.15) | NA | [110] |
|  |  |  | Asian | 2 | 344 | 272 | 0.52(0.25-1.11) | 0.56 |  |
|  |  | CR vs RR | European | 3 | 271 | 570 | 1.45(1.00-2.12) | NA |  |
|  |  |  | Asian | 2 | 179 | 92 | 0.78(0.36-1.68) | 0.26 |  |
|  |  | **Dominant** | **European** | **3** | **497** | **983** | **1.06(0.85-1.33)** | **0.92** |  |
|  |  |  | **Asian** | **2** | **497** | **354** | **0.69(0.50-0.95)** | **NA** |  |
|  |  | Recessive | European | 3 | 497 | 983 | 1.45(1.01-2.08) | NA |  |
|  |  |  | Asian | 2 | 497 | 354 | 0.62(0.30-1.29) | 0.09 |  |
