# Supplementary material for: Ethnic Background and Genetic Variation in the Evaluation of Cancer Risk: A Systematic Review
Source: PLoS One. 2014 Jun 5;9(6):e97522. doi: 10.1371/journal.pone.0097522 (PMC4046957; doi:10.1371/journal.pone.0097522)
Supplement: Tables S6 — The association of the assessed variations with risk of gastric cancer [111]–[116]. (DOCX) [file pone.0097522.s009.docx]

Table S6 Associations with lung cancer

| **Gene** | **SNP** | **Model** | **Ethnicity** | **# of studies** | **# of cases** | **# of controls** | **Odd's Ratio** | **Power** | **Reference** |
| --- | --- | --- | --- | --- | --- | --- | --- | --- | --- |
| COX-2 | rs5275 | RR vs CC | European | 2 | 1299 | 1436 | 0.87(0.72-1.05) | NA | [111] |
|  |  |  | Asian | 3 | 855 | 1046 | 0.71(0.44-1.17) | NA |  |
|  |  | **Dominant** | **European** | **2** | **2368** | **2681** | **0.91(0.81-1.02)** | **0.87** |  |
|  |  |  | **Asian** | **3** | **1620** | **1263** | **0.84(0.72-0.98)** | **NA** |  |
| CYP1B1 | rs1056836 | **RR vs CC** | **European** | **4** | **702** | **1101** | **1.570 (1.049-2.350)** | **NA** | [112] |
|  |  |  | **Asian** | **4** | **708** | **709** | **1.417(0.805-2.494)** | **0.98** |  |
|  |  |  | **African American** | **2** | **89** | **91** | **2.004(0.109-36.851)** | **0.08** |  |
|  |  | CC vs CR | European | 4 | 1050 | 1662 | 1.400(0.947-2.068) | NA |  |
|  |  |  | Asian | 4 | 940 | 942 | 1.099(0.817-1.479) | NA |  |
|  |  |  | African American | 2 | 58 | 57 | 2.118(0.132-33.951) | NA |  |
| ERCC2 | rs13181 | **RR vs CC** | **European** | **9** | **1791** | **2033** | **1.25 (1.08–1.45)** | **NA** | [113] |
|  |  |  | **Asian** | **8** | **2724** | **2735** | **1.09 (0.69–1.71)** | **0.9** |  |
|  |  |  | **African American** | **2** | **245** | **325** | **1.17 (0.65–2.10)** | **0.26** |  |
|  |  | CC vs CR | European | 9 | 2816 | 3268 | 1.06 (0.96–1.18) | NA |  |
|  |  |  | Asian | 8 | 3199 | 3171 | 1.12 (0.87–1.42) | NA |  |
|  |  |  | African American | 2 | 385 | 487 | 1.10 (0.96–1.26) | NA |  |
|  |  | Dominant | European | 9 | 3305 | 3737 | 1.22 (1.06–1.40) | NA |  |
|  |  |  | Asian | 8 | 3241 | 3210 | 1.08 (0.69–1.68) | 0.87 |  |
|  |  |  | African American | 2 | 408 | 514 | 1.10 (0.62–1.95) | 0.32 |  |
|  |  | Recessive | European | 9 | 3305 | 3737 | 1.10 (1.00–1.22) | NA |  |
|  |  |  | Asian | 8 | 3241 | 3210 | 1.11 (0.86–1.43) | 0.06 |  |
|  |  |  | African American | 2 | 408 | 514 | 1.17 (0.90–1.53) | 0.05 |  |
| hOGG1 | rs1052133 | RR vs CC | European | 10 | 2781 | 3287 | 0.97(0.71-1.34) | 0.85 | [114] |
|  |  |  | Asian | 10 | 2042 | 2144 | 1.17(1.02-1.35) | NA |  |
|  |  | CC vs CR | European | 10 | 3906 | 4574 | 0.98(0.84-1.15) | 0.85 |  |
|  |  |  | Asian | 10 | 2798 | 3195 | 1.14(1.01-1.28) | NA |  |
|  |  | **Dominant** | **European** | **10** | **4134** | **4854** | **0.97(0.82-1.14)** | **0.9** |  |
|  |  |  | **Asian** | **10** | **3900** | **4208** | **1.15(1.03-1.29)** | **NA** |  |
| MDM2 | rs2279744 | **RR vs CC** | **European** | **4** | **1925** | **1763** | **1.11(0.98-1.47)** | **0.99** | [115] |
|  |  |  | **Asian** | **3** | **1191** | **1537** | **1.34(1.01-1.79)** | **NA** |  |
|  |  |  | **African** | **1** | **113** | **208** | **0.73(0.14-3.83)** | **0.19** |  |
|  |  | CC vs CR | European | 4 | 2995 | 2885 | 0.92(0.83-1.02) | 0.92 |  |
|  |  |  | Asian | 3 | 1742 | 2362 | 1.20(1.05-1.37) | NA |  |
|  |  |  | African | 1 | 131 | 250 | 0.78(0.44-1.37) | 0.11 |  |
|  |  | Dominant | European | 4 | 3525 | 3338 | 0.95(0.86-1.05) | 1 |  |
|  |  |  | Asian | 3 | 2405 | 3085 | 1.26(1.11-1.43) | NA |  |
|  |  |  | African | 1 | 133 | 255 | 0.77(0.45-1.34) | 0.15 |  |
|  |  | Recessive | European | 4 | 3525 | 3338 | 1.11(0.97-1.28) | NA |  |
|  |  |  | Asian | 3 | 2405 | 3085 | 1.21(0.97-1.51) | NA |  |
|  |  |  | African | 1 | 133 | 255 | 0.76(0.15-3.99) | NA |  |
| TP53 | rs1042522 | **RR vs CC** | **European** | **10** | **2021** | **2522** | **0.986(0.742-1.310)** | **1** | [116] |
|  |  |  | **Asian** | **8** | **1962** | **1685** | **1.359(1.206-1.613)** | **NA** |  |
|  |  | Dominant | European | 10 | 3359 | 3953 | 1.180(1.029-1.353) | NA |  |
|  |  |  | Asian | 8 | 3254 | 3350 | 1.109(1.000-1.228) | NA |  |
