# Supplementary material for: Ethnic Background and Genetic Variation in the Evaluation of Cancer Risk: A Systematic Review
Source: PLoS One. 2014 Jun 5;9(6):e97522. doi: 10.1371/journal.pone.0097522 (PMC4046957; doi:10.1371/journal.pone.0097522)
Supplement: Tables S7 — The association of the assessed variations with risk of prostate cancer [117]–[121]. (DOCX) [file pone.0097522.s010.docx]

Table S7 Associations with prostate cancer

| **Gene** | **SNP** | **Model** | **Ethnicity** | **# of studies** | **# of cases** | **# of controls** | **Odd's Ratio** | **Power** | **Reference** |
| --- | --- | --- | --- | --- | --- | --- | --- | --- | --- |
| CYP17 | rs743572 | **C vs R' OR 'Allele'** | **European** | **7** | **1948** | **2110** | **1.04(0.92-1.18)** | **1.00** | [117] |
|  |  |  | **Asian** | **2** | **375** | **545** | **1.06(0.66-1.71)** | **0.65** |  |
|  |  |  | **African** | **3** | **113** | **134** | **1.56(1.07-2.28)** | **NA** |  |
| FGFR4 | rs351855 | C vs R' OR 'Allele' | European | 3 | 1935 | 1787 | 1.21(1.00-1.47) | NA | [118] |
|  |  |  | Asian | 1 | 492 | 344 | 1.24(1.02-1.51) | NA |  |
|  |  |  | African | 2 | 191 | 174 | 1.15(0.73-1.82) | 0.13 |  |
|  |  | **RR vs CC** | **European** | **3** | **1092** | **1084** | **1.40(0.80-2.45)** | **1.00** |  |
|  |  |  | **Asian** | **1** | **296** | **192** | **1.52(1.05-2.22)** | **NA** |  |
|  |  |  | **African** | **2** | **146** | **138** | **2.17(0.20-23.14)** | **0.33** |  |
|  |  | Dominant | European | 3 | 1935 | 1787 | 1.23(1.08-1.40) | NA |  |
|  |  |  | Asian | 1 | 492 | 344 | 1.15(0.86-1.54) | 0.28 |  |
|  |  |  | African | 2 | 191 | 174 | 1.11(0.66-1.86) | 0.13 |  |
|  |  | Recessive | European | 3 | 1935 | 1787 | 1.26(0.72-2.19) | 0.46 |  |
|  |  |  | Asian | 1 | 492 | 344 | 1.53(1.10-2.14) | NA |  |
|  |  |  | African | 2 | 191 | 174 | 2.21(0.18-26.83) | 0.07 |  |
| SOD2 | rs4880 | RR vs CC | European | 8 | 1587 | 2283 | 1.18(0.97-1.44) | NA | [119] |
|  |  |  | African | 3 | 78 | 321 | 1.46(0.49-4.34) | NA |  |
|  |  |  | mixed | 1 | 101 | 79 | 1.50(0.83-2.72) | NA |  |
|  |  | CC vs CR | European | 8 | 2383 | 3438 | 1.12(1.00-1.25) | NA |  |
|  |  |  | African | 3 | 152 | 554 | 1.25(0.85-1.83) | 0.09 |  |
|  |  |  | mixed | 1 | 127 | 136 | 0.87(0.52-1.48) | NA |  |
|  |  | **Dominant** | **European** | **8** | **3205** | **4541** | **1.14(1.02-1.26)** | **NA** |  |
|  |  |  | **African** | **3** | **182** | **672** | **1.19(0.83-1.73)** | **0.11** |  |
|  |  |  | **mixed** | **1** | **187** | **175** | **1.06(0.64-1.73)** | **NA** |  |
|  |  | Recessive | European | 8 | 3205 | 4541 | 1.09(0.98-1.21) | 0.79 |  |
|  |  |  | African | 3 | 182 | 672 | 1.23(0.44-3.49) | 0.13 |  |
|  |  |  | mixed | 1 | 187 | 175 | 1.65(1.03-2.64) | NA |  |
| SRD5A2 | rs523349 | RR vs CC | European | 18 | 3561 | 4508 | 1.16(0.96-1.40) | NA | [120] |
|  |  |  | Asian | 5 | 452 | 584 | 0.92(0.71-1.19) | NA |  |
|  |  |  | African | 4 | 451 | 321 | 1.14(0.75-1.74) | NA |  |
|  |  | **Dominant** | **European** | **18** | **6217** | **7564** | **1.11(1.03-1.19)** | **NA** |  |
|  |  |  | **Asian** | **5** | **842** | **1133** | **0.88(0.72-1.08)** | **0.17** |  |
|  |  |  | **African** | **4** | **755** | **866** | **1.03(0.84-1.26)** | **0.18** |  |
|  |  | Recessive | European | 18 | 6217 | 7564 | 1.07(0.88-1.29) | NA |  |
|  |  |  | Asian | 5 | 842 | 1133 | 0.98(0.79-1.22) | NA |  |
|  |  |  | African | 4 | 755 | 866 | 1.14(0.76-1.72) | NA |  |
| XRCC1 | rs25487 | **RR vs CC** | **European** | **4** | **533** | **506** | **1.03(0.76-1.41)** | **0.93** | [121] |
|  |  |  | **Asian** | **4** | **414** | **472** | **1.55(1.02-2.33)** | **NA** |  |
|  |  | Dominant | European | 4 | 941 | 879 | 1.04(0.86-1.25) | NA |  |
|  |  |  | Asian | 4 | 669 | 762 | 1.17(0.93-1.46) | NA |  |
|  |  | Recessive | European | 4 | 941 | 879 | 1.02(0.76-1.36) | 0.20 |  |
|  |  |  | Asian | 4 | 669 | 762 | 1.43(1.02-2.00) | NA |  |
